# Delayed kernels for longitudinal survival analysis and dynamic prediction

**DOI:** 10.1177/09622802241275382

**Published:** 2024-08-30

**Authors:** Annabel Louisa Davies, Anthony CC Coolen, Tobias Galla

**Affiliations:** 1 Department of Physics and Astronomy, University of Manchester, UK; 2 Department of Population Health Sciences, Bristol Medical School, 1980 University of Bristol, UK; 3 Department of Biophysics, Radboud University, the Netherlands; 4 Saddle Point Science Ltd, UK; 5 Instituto de Física Interdisciplinar y Sistemas Complejos, IFISC (CSIC-UIB), Campus Universitat Illes Balears, Palma de Mallorca, Spain

**Keywords:** Dynamic prediction, joint modelling, landmarking, survival analysis, time-dependent covariates, weighted cumulative effects

## Abstract

Predicting patient survival probabilities based on observed covariates is an important assessment in clinical practice. These patient-specific covariates are often measured over multiple follow-up appointments. It is then of interest to predict survival based on the history of these longitudinal measurements, and to update predictions as more observations become available. The standard approaches to these so-called ‘dynamic prediction’ assessments are joint models and landmark analysis. Joint models involve high-dimensional parameterizations, and their computational complexity often prohibits including multiple longitudinal covariates. Landmark analysis is simpler, but discards a proportion of the available data at each ‘landmark time’. In this work, we propose a ‘delayed kernel’ approach to dynamic prediction that sits somewhere in between the two standard methods in terms of complexity. By conditioning hazard rates directly on the covariate measurements over the observation time frame, we define a model that takes into account the full history of covariate measurements but is more practical and parsimonious than joint modelling. Time-dependent association kernels describe the impact of covariate changes at earlier times on the patient’s hazard rate at later times. Under the constraints that our model (a) reduces to the standard Cox model for time-independent covariates, and (b) contains the instantaneous Cox model as a special case, we derive two natural kernel parameterizations. Upon application to three clinical data sets, we find that the predictive accuracy of the delayed kernel approach is comparable to that of the two existing standard methods.

## 1. Introduction

Survival analysis is a well-established field of medical statistics that involves modelling the probability of survival until some specified irreversible event such as death or the onset of disease. Of particular clinical interest is the prediction of patient-specific survival based on a set of observed biomarkers or ‘covariates’.^1^ Such predictions aid clinicians in making treatment and testing decisions and provide personalized information for patients about their health.^2^

Cox’s proportional hazards (PH) model^3^ remains the most widely used model in survival analysis.^4,5^ In this context, survival is assumed to depend on a set of covariates, $z=\{z_{\mu };\mu =1,\ldots p\}$ , measured at some baseline time. The hazard, h(t) , is the instantaneous event rate, defined as follows:

(1) $$h(t|z)=h_{0}(t)e^{\sum_{\mu =1}^{p}\beta_{\mu }z_{\mu }}$$

where the $\beta_{\mu }$ (with μ=1,…,p ) are the so-called association parameters. The baseline hazard function, $h_{0}(t)$ , is the value of the hazard for covariate values $z_{\mu }=0$ ∀μ . The name ‘proportional hazards’ refers to the fact that, due to the exponential form of the hazard function, the effect of each covariate on the hazard is multiplicative. In this work, we will call the model in equation (1) the ‘standard Cox model’.

Survival prediction in the standard Cox model is based on the survival function

(2) $$S(t|z)=e^{-\int_{0}^{t}h(t^{\prime }|z)dt^{\prime }}$$

that describes the probability that an individual with covariate values $z=\{z_{\mu }\}$ experiences the event after time t .

In reality, covariates are often measured repeatedly over time. This means that multiple observations of time-dependent covariates $\left\{z_{\mu }(t)\right\}$ are made for any particular patient. A simple extension to the standard Cox model involves modelling the hazard rate as dependent on the instantaneous value of the covariates,^6–8^ that is

(3) $$h(t|z(t))=h_{0}(t)e^{\sum_{\mu =1}^{p}\beta_{\mu }z_{\mu }(t)}$$

where we have written z(t) for the collection of instantaneous observations $z_{\mu }(t)$ , μ=1,…,p . We refer to equation (3) as the ‘instantaneous Cox model’.

However, in practice, one does not have access to the full covariate trajectories $z_{\mu }(t)$ . Instead, observations are made at discrete follow-up times until some subject-specific final observation time. Since we do not have access to covariate measurements after this time, we cannot make predictions about future survival probabilities based on equation (3). Due to these difficulties, survival predictions are commonly evaluated using a standard Cox model with the baseline measurement as the only predictor.^2^ By not including the follow-up observations, this standard practice discards a potentially considerable proportion of the available patient data.

Recently, there has been much interest in so-called “dynamic prediction”.^9,10^ These methods aim to make survival predictions based on the longitudinal history of biomarker data and update these predictions as more data becomes available. Such analysis is clinically valuable as it allows patients and clinicians to review disease progression over time and update the prognosis at each follow-up visit.^11^ Currently, there are two main approaches to dynamic prediction; joint modelling and landmarking. Landmarking was an early approach to the problem,^12^ whereby a standard Cox model is fitted to patients in the original data set who are still at risk at the time point of interest, using their most recent covariate measurements. More recently, joint modelling (JM) has become an established method.^13–16^ Here one models the time-dependent covariate trajectory using a parameterized longitudinal model, and this complete trajectory is then inserted into an instantaneous Cox-type survival model. A joint likelihood of the longitudinal and survival sub-models is constructed, and the model parameters are estimated via maximum likelihood (ML) or Bayesian inference.

Both methods have limitations. In particular, joint models are demanding both conceptually and computationally. Correctly modelling the longitudinal trajectories can be difficult when patient measurements exhibit varied non-linear behaviour^11^ and mis-specification of this trajectory has been found to lead to bias.^17^ Furthermore, the number of model parameters increases rapidly with the inclusion of multiple longitudinal markers. This means that many software packages cannot handle more than one longitudinal covariate,^18–20^ and those that can quickly become computationally intensive.^21,22^ For these reasons, the landmarking model is often seen as the only practical option.^11^ However, the relative simplicity of the landmarking approach comes with its own drawbacks. By using only the ‘at risk’ data set to make predictions at a certain time (discarding patients who had an event before the landmark time), landmarking makes use of only a subset of the available data. In standard landmarking approaches, the history of the covariate values are not taken into account directly, and a new model must be fitted every time one wishes to update the predictions.

In this work, we present a new approach to dynamic prediction that conceptually and in terms of computational complexity lies somewhere in between the JM and landmarking methods. Rather than modelling the covariate trajectory at future times, as in the JM approach, we model the probability of survival conditioned directly on the observed covariates measured from the baseline time up to a subject-specific final observation time. Unlike the landmark approach, a single model is fitted to all of the available data, using the full history of the covariate values. We do, however, maintain well-established and desirable features of the Cox model, so that our model contains the instantaneous Cox model as a special case, and reduces automatically to the standard Cox model for covariates that are observed to be fixed over time. Within these constraints, we define time-dependent parametric association kernels, $\beta_{\mu }(t,t^{\prime },s)$ , that describe the impact of changes of covariate μ at time $t^{\prime }$ on patient risk at some later time t . The kernel can also depend on the final observation time s for the patient. Building on ideas from weighted cumulative exposure models,^23,24^ these kernels allow us to assign smaller effects to covariates that were measured further in the past. We refer to our method as the ‘delayed kernel’ approach.

The remainder of this article is set out as follows. In Section 2, we introduce the motivating data sets. In Section 3, we then provide details of the dynamic prediction models. We begin by describing the longitudinal and time-to-event data, and briefly outline the standard methods: JM (Section 3.2) and landmarking (Section 3.3). In Section 3.4, we introduce the delayed kernel (DK) approach. We start by defining the hazard rate conditioned on the observed data and then develop two natural parameterizations for the association kernels that meet our requirements. We outline the ML method for parameter estimation for these models and show how the DK approach can be used to make dynamic predictions. In Section 3.5, we describe a simple simulation study to aid the interpretation of our model parameters. Via application to the real data sets, in Section 4, we compare the performance of the delayed kernel approach to the standard methods using an established measure of predictive accuracy. Finally, we discuss and summarize our results in Section 5.

## 2. Motivating data sets

In our work, we will assess the predictive capabilities of the different models for dynamic prediction using three clinical data sets that contain both longitudinal covariate measurements and time-to-event data. All three data sets are publicly available in the JMbayes package^25^ (and its update JMbayes2^26^), and were used by Rizopoulos^16^ to illustrate the JM method.

### 2.1. Primary biliary cirrhosis (PBC)

**Figure 1.**
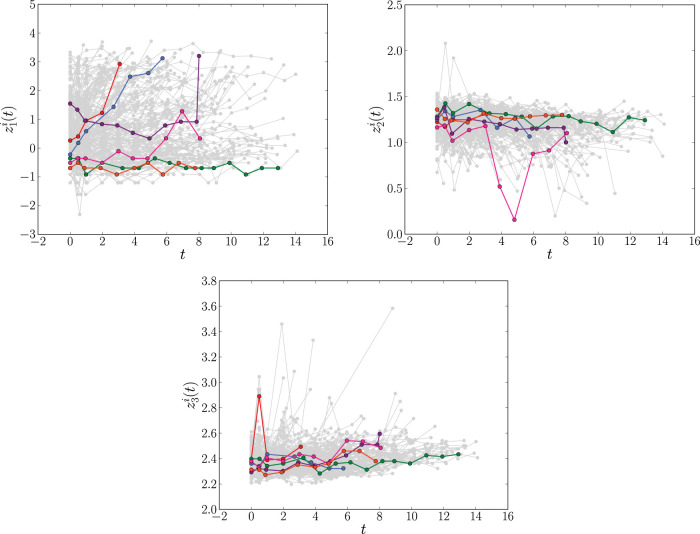
The longitudinal profiles of the time-dependent covariates log serum bilirubin ( $z_{1}^{i}(t)$ ), log serum albumin ( $z_{2}^{i}(t)$ ) and log prothrombin time ( $z_{3}^{i}(t)$ ) for the N=312 patients ( i=1,…,N ) in the primary biliary cirrhosis (PBC) data set described in Section 2.1. For clarity, the trajectories of six individuals are highlighted. Time, t , on the x -axis is measured in years.

The first motivating data set is from a study conducted by the Mayo Clinic from 1974 to 1984 on patients with primary biliary cirrhosis (PBC), a progressive chronic liver disease.^27^ We will refer to this as the PBC data. The study involved N=312 patients who were randomly assigned either a placebo (154 patients) or the D-penicillamine treatment (158 patients). Time-to-event data is available for the outcome of interest (death) or the censoring event (either the time at which the patient receives a liver transplant or the final follow-up time at which they were still alive). By the end of follow-up, 140 patients had died, 29 had received a transplant and 143 were still alive. As well as baseline covariate measurements such as age at baseline and gender, multiple longitudinal biomarker measurements were collected for each patient over an average number of 6.2 visits from study entry to some subject-specific final observation time (prior to their event time). While the original aim of the study was to investigate the effect of the drug D-penicillamine, no effect was found and the data has since been used to study the progression of the disease based on longitudinal biomarkers.^28^ With this in mind, we include age at baseline as our only fixed covariate and focus on the longitudinal covariates log serum bilirubin, log serum albumin and log prothrombin time, which have previously been found to be indicators of patient survival.^28^ Serum bilirubin and serum albumin indicate concentrations of these substances in the blood, measured in mg/dL and g/dL, respectively. Prothrombin time measures the time (in seconds) it takes for blood to clot in a sample. Time series of these three longitudinal biomarkers are plotted in Figure 1.

**Figure 2.**
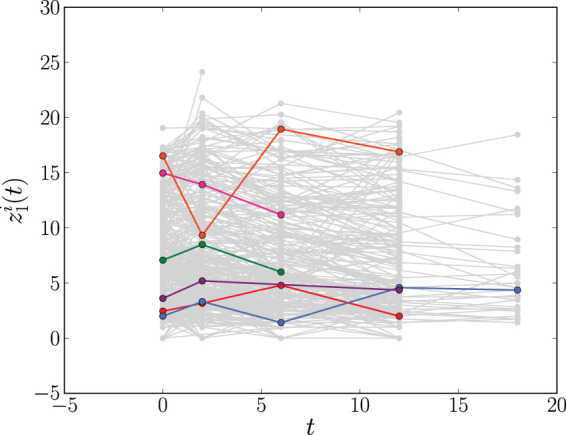
The longitudinal profiles of CD4 count ( $z_{1}^{i}(t)$ ) in the N=467 patients ( i=1,…,N ) in the AIDS data set in Section 2.2. For clarity, the trajectories of six individuals are highlighted. Time, t , is measured in months. CD4 = clusters of differentiation 4; AIDS = acquired immunodeficiency syndrome.

### 2.2. Acquired immunodeficiency syndrome (AIDS)

The second data set involves N=467 HIV-infected patients who had failed to respond, or were intolerant to, zidovudine (previously called ‘azidothymidine’) therapy (AZT).^29^ The aim of the study was to compare two antiretroviral drugs, didanosine (ddI) and zalcitabine (ddC). Patients were randomly assigned one of these drugs at baseline. Patients’ CD4 cell counts were recorded at baseline and follow-up measurements were planned at 2, 6, 12, and 18 months. CD4 cells are white blood cells that fight infections. A decrease in the number of CD4 cells over time indicates a worsening of the immune system and higher susceptibility to infection. Therefore, the number of CD4 cells in a blood sample is an important marker of immune strength and hence a covariate of interest in HIV-infected patients. In line with previous analyses of this data,^16,30^ we use the square root of the CD4 count as our longitudinal covariate. For brevity, we will refer to this simply as the CD4 count. By the end of the study, 118 patients had died, and the time-to-event (death) or censoring was recorded for all patients. Final observation times ( $s_{i}\in [0,2,6,12,18]$ months) were always less than their corresponding event times, such that there is a time gap between when a subject was last observed and when they experienced an event. Following Guo and Carlin,^30^ we included, in addition to the longitudinal CD4 counts and the patients’ drug group, also three other binary fixed covariates in our analysis: gender, PrevOI (previous opportunistic infection—AIDS diagnosis—at study entry), and stratum (whether the patient experienced AZT failure or AZT intolerance). We will refer to this data as the AIDS data set. The longitudinal profiles of the CD4 count for all patients are plotted in Figure 2.

### 2.3. Liver cirrhosis

**Figure 3.**
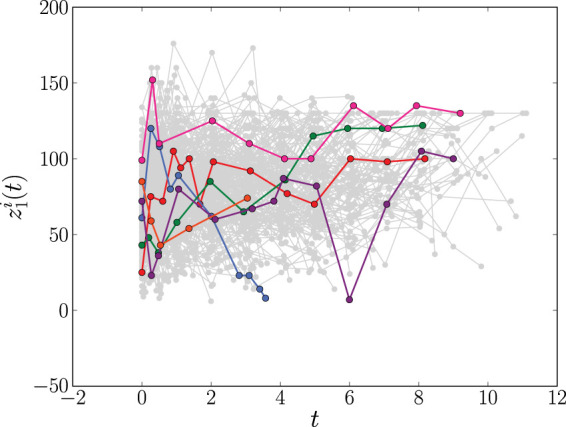
The longitudinal profiles of the prothrombin index ( $z_{1}^{i}(t)$ ) as measured in the N=488 patients ( i=1,…,N) in the Liver data set described in Section 2.3. For clarity, the trajectories of six individuals are highlighted. Time, t , is measured in years.

The third data set is from a trial conducted between 1962 and 1974, involving N=488 patients with liver cirrhosis, a general term including all forms of chronic diffuse liver disease.^31^ We call this the Liver data set. At baseline, 251 patients were randomly assigned a placebo and 237 were assigned treatment with the drug prednisone. Follow-up appointments were scheduled at 3, 6 and 12 months and then yearly thereafter, though actual follow-up times varied considerably. At these follow-up appointments, multiple longitudinal biomarkers were measured. However, only the prothrombin index measurements are available from the JMbayes package. This is a measure of liver function based on a blood test of coagulation factors produced by the liver. For reproducibility, and following previous analyses of the Liver data set,^16,32^ we include the prothrombin index as our only time-dependent biomarker. The drug group is included as a fixed baseline covariate. By the end of the study, 150 prednisone-treated, and 142 placebo-treated patients had died. Their time-to-event data was recorded. Of the 488 subjects, 120 were observed until their event time while all others were observed until some subject-specific final observation time before their event time. Figure 3 shows the longitudinal prothrombin measurements for all patients.

## 3. Dynamic prediction models

### 3.1. Setup and notation

In this work, we consider longitudinal survival data of the form $D=\{T_{i},\delta_{i},Z^{i};i=1,\ldots ,N\}$ where $T_{i}=\min(T_{i}^{*},C_{i})$ is the observed event time of individual i with $T_{i}^{*}$ denoting the true event time and $C_{i}$ denoting the censoring time. The event indicator $\delta_{i}=I(T_{i}^{*}\leq C_{i})$ is equal to 1 when the true event time is observed and 0 when it is censored. Throughout this article, we use the indicator function I(A) , defined as I(A)=1 if A holds, and I(A)=0 otherwise. We use the roman indices i,j,k to label individuals in the data set. $Z^{i}=\{z_{\mu }^{i}(t_{i\ell });\;\mu =1,\ldots ,p,\;\ell =1,\ldots ,n_{i},\;t_{i\ell }\in [0,s_{i}]\}$ denotes the set of time-dependent covariate observations of individual i . Individual i has $n_{i}$ measurements of p longitudinal covariates from time t=0 up to some subject-specific final observation time $s_{i}\leq T_{i}$ . These measurements are taken at discrete (subject-specific) observation times, $t_{i\ell }$ , $\ell =1,\ldots ,n_{i}$ , where $t_{i1}=0$ and $t_{in_{i}}=s_{i}$ . We write $Z_{\left[0,s_{i}\right]}^{i}=\{z_{\mu }^{i}(t);\;\mu =1\ldots p,\;t\in [0,s_{i}]\}$ for the ‘true’ (but non-accessible) continuous trajectories of the p covariates over the interval $t\in [0,s_{i}]$ for individual i . We develop our theory based on the assumption that we have access to these trajectories $Z_{\left[0,s_{i}\right]}^{i}$ . As we will see later, we estimate $Z_{\left[0,s_{i}\right]}^{i}$ from the discrete observations $Z^{i}$ .

We are interested in predicting survival probabilities for some new subject with longitudinal measurements $Z=\{z_{\mu }(t_{\ell });\;\mu =1,\ldots ,p,\;\ell =1,\ldots ,n,\;t_{\ell }\in [0,s]\}$ . The quantity we wish to estimate is the probability that this subject survives until some future time u>s , conditional on their survival to s , and on their covariate observations up to s . That is

(4) $$\pi (u|Z_{\left[0,s\right]},s)=\mathrm{Pr}(T^{*}\geq u|\;T^{*}>s,Z_{\left[0,s\right]},D)$$

The quantity $\pi (u|Z_{\left[0,s\right]},s)$ is referred to as a “dynamic predictor” due to the fact that it can be updated as more measurements become available at later times.^2,33^

### 3.2. Joint models

In JM one specifies two model components: a longitudinal model for the trajectory of the time-dependent covariates, and a survival model which relates to the covariate trajectory via shared parameters. In the JMbayes and JMbayes2 packages, joint models are fitted using Bayesian inference by specifying a joint likelihood distribution for the two model components and a set of prior distributions on the model parameters. Details of this package and the JM framework we follow are described by Rizopoulos.^16,25^ In this section, we briefly outline the model.

#### 3.2.1. Longitudinal modelling component

Mixed-effects models are typically specified for the longitudinal covariate trajectories, where it is assumed that the observed value $z_{\mu }(t)$ of the covariate at time t deviates from the true (unobserved) value $m_{\mu }(t)$ by an amount $\varepsilon_{\mu }(t)$ . The error terms $\varepsilon_{\mu }(t)$ of all subjects are assumed to be statistically independent, and normally distributed with variance,

(5) $$\begin{array}{rl} & z_{\mu }^{i}(t)=m_{\mu }^{i}(t)+\varepsilon_{\mu }^{i}(t),\;\;\;\;m_{\mu }^{i}(t)=x_{\mu }^{i⊤}(t)\eta_{\mu }+y_{\mu }^{i⊤}(t)b_{\mu }^{i}\\ & b^{i}∼N(0,D),\;\varepsilon_{\mu }^{i}(t)∼N(0,\sigma_{\mu }^{2}) \end{array}$$

Between-subject variability is modelled via estimation of subject-specific random effects $b_{\mu }^{i}$ , whereas effects that are shared between all subjects are modelled by the fixed effects $\eta_{\mu }$ . The vectors $x_{\mu }^{i⊤}(t)$ and $y_{\mu }^{i⊤}(t)$ denote the design vectors for these fixed and random effects respectively. For multivariate models, one can allow for association between the different longitudinal markers via their corresponding random effects. In particular, we assume that the complete vector of random effects $b^{i}=(b_{1}^{i⊤},\ldots ,b_{p}^{i⊤}{)}^{⊤}$ follows a multivariate normal distribution with mean zero and variance-covariance matrix D that describes the correlations between and variances of the random effects. For details, we refer to references.^2,16,21,34,35^

#### 3.2.2. Survival modelling component

The hazard at time t is assumed to depend on the value of the longitudinal covariate at time t without measurement error, that is,

(6) $$h^{\mathrm{JM}}(t|M_{\left[0,t\right]})=h_{0}(t)\exp\;\left\{\sum_{\mu =1}^{p}\alpha_{\mu }m_{\mu }(t)\right\}$$

where $M_{\left[0,t\right]}=\{m_{\mu }(t^{\prime });\;\mu =1,\ldots ,p,\;t^{\prime }\in [0,t]\}$ denotes the history of the ‘true’ (unobserved) longitudinal covariates up to time t . Note that in equation (6) the hazard rate depends only on the instantaneous values of the covariates, but this can be generalized as briefly highlighted below. Unlike in the Cox model, the baseline hazard, $h_{0}(t)$ cannot be expressed analytically in terms of the other model parameters during the ML procedure, but must instead be specified. Often this is done using a flexible parametric model, for example, using penalized spline functions.^25^ Dependence of the hazard function on time-independent covariates $\left\{\zeta_{\nu };\;\nu =1,\ldots ,q\right\}$ can be included through an additional term $\sum_{\nu =1}^{q}\gamma_{\nu }\zeta_{\nu }$ in the exponent in equation (6), where $\gamma_{\nu }$ is the association parameter for fixed covariate ν . Alternative extensions allow the hazard to depend on the slope of the covariate trajectory, or on its cumulative effect, by replacing the term $\alpha_{\mu }m_{\mu }(t)$ with $\alpha_{\mu }^{\left(1\right)}m_{\mu }(t)+\alpha_{\mu }^{\left(2\right)}\frac{d}{dt}m_{\mu }(t)$ or with $\alpha_{\mu }\int_{0}^{t}m_{\mu }(t^{\prime })dt^{\prime }$ , respectively.^2,16^

It has also been proposed to introduce a weight function to capture cumulative effects, writing $\alpha_{\mu }\int_{0}^{t}w_{\mu }(t-t^{\prime })m_{\mu }(t^{\prime })dt^{\prime }$ , with kernels $w_{\mu }(t-t^{\prime })$ defined such that earlier covariate values have a smaller effect on the hazard than recent values.^16,36^ This idea is connected to the concept of ‘weighted cumulative exposure’ (WCE).^23,24^ WCE models were developed in etiological research to describe the complex cumulative effect of time-dependent ‘exposure’, for example, to a drug, on health outcomes.^37^ In a survival context, these models rely on continuous knowledge of the exposure all the way up to the event time, and have hence been used almost exclusively for measuring the effects of external exposures such as treatments or environmental factors.^38^ JM allows the principles of WCE to be used for any longitudinal covariate. Through the prediction of future covariate trajectories, it is also possible for these ideas to be integrated into dynamic predictions.^36^ As we will explain later, we build on the principles of WCE to develop our DK approach.

#### 3.2.3. Dynamic prediction

Finally, in the JM framework we use, the quantity $\pi (u|Z_{\left[0,s\right]},s)$ is estimated using a Bayesian approach, with posterior parameter distribution $p(\theta_{\mathrm{JM}}|D)$ and where $\theta_{\mathrm{JM}}$ is the vector of all the model parameters in the joint model. This leads to the estimator

(7) $${\hat{\pi }}^{\mathrm{JM}}(u|Z_{\left[0,s\right]},s)=\int \mathrm{Pr}(T^{*}\geq u|\;T^{*}>s,Z_{\left[0,s\right]},\theta_{\mathrm{JM}})p(\theta_{\mathrm{JM}}|D)\;d\theta_{\mathrm{JM}}$$

The parameter average in equation (7) can generally not be evaluated analytically, and is computed via Monte Carlo methods. Again, we refer to references^2,16,34^ for details.

#### 3.2.4. Limitations

Joint models have undergone much development over recent years, with various extensions making the approach flexible in a range of different scenarios. However, the JM approach requires the ability to correctly specify both the longitudinal and survival model. This can involve modelling assumptions which are not always easy to verify. Indeed, simulations have demonstrated that the JM approach is biased under mis-specification of the longitudinal model.^17^ In addition, as more longitudinal outcomes are included, the dimensionality of the random effects increases, and fitting the joint model becomes computationally intensive. Depending on the longitudinal model specified, it can be difficult to include more than three or four longitudinal covariates.^11,21^ This is amplified when cumulative or weighted cumulative effects are used in the survival model (as numerical integration of the longitudinal model is required). As a result, there are cases where joint models are not a viable option and, instead, one must rely on approaches such as landmarking.^11^

### 3.3. Landmarking

#### 3.3.1. Description of the landmarking procedure

The landmarking approach to dynamic prediction is based on the standard Cox model.^1,12,33^ Upon denoting with $R(\upsilon )=\{i:\;T_{i}>\upsilon \}$ the set of individuals in the original data set who are still at risk at time υ , the landmarking model assumes that for a subject in the risk set R(υ) the distribution of survival times, conditioned on the covariate measurements $\left\{z_{\mu }^{i}(\upsilon )\right\}$ at that time, follows a standard Cox model.^11^ In general one does not have covariate measurements for all individuals at time υ . Instead, one uses for each individual the last observation $\left\{{\tilde{z}}_{\mu }(\upsilon ),\;\mu =1,\ldots ,p\right\}$ of the covariates before time υ , and treats these as fixed covariates in a standard Cox model with υ as the baseline time. That is, for the so-called “landmark time” υ one defines the hazard rate at times t>υ as

(8) $$h^{\mathrm{LM}}(t|Z,\upsilon )=h_{0}(t|\upsilon )\exp\;\left\{\sum_{\mu =1}^{p}\alpha_{\mu }(\upsilon ){\tilde{z}}_{\mu }(\upsilon )\right\}$$

The baseline hazard function $h_{0}(t|\upsilon )$ is unspecified and is estimated as in standard Cox models, via partial likelihood arguments, or via functional maximization of the data log-likelihood. Subsequently, the association parameters are estimated. This procedure is carried out for each choice of the landmark time υ , and leads to the Breslow estimator^39^ ${\hat{h}}_{0}(t|\upsilon )$ and the association parameters ${\hat{\alpha }}_{\mu }(\upsilon )$ . The main difference compared to standard Cox models is the dependence of association parameters and the baseline hazard function on the landmark time υ .

To estimate the quantity $\pi (u|Z_{\left[0,s\right]},s)$ , the landmark time υ in equation (8) is set equal to s . Once this model is fitted, survival prediction to time u>s is performed using the standard Cox survival probability

(9) $${\hat{\pi }}^{\mathrm{LM}}(u|Z_{\left[0,s\right]},s)=\exp\;\{-e^{\sum_{\mu =1}^{p}{\hat{\alpha }}_{\mu }(s){\tilde{z}}_{\mu }(s)}\int_{s}^{u}{\hat{h}}_{0}(t^{\prime }|s)dt^{\prime }\}$$

#### 3.3.2. Limitations

Landmarking is computationally and conceptually much simpler than the JM approach. For data sets with multiple longitudinal covariates, disparate non-linear covariate trajectories or categorical time-dependent covariates, landmarking is often the preferred approach.^11^ However, it also has limitations. For example, the standard model focuses only on the most recent value observed before time υ , and does not account for the earlier history of covariates. Extensions to the standard approach which use some more involved summary of the covariate history (e.g. a weighted average of values up to the landmark time) are possible. However, even in this scenario, data from individuals who experience the event before time υ is not used for the parameter estimation at landmark time υ . Therefore, the landmark approach uses only a subset of the available data. Furthermore, a new Cox model has to be specified and fitted for each landmark time. Therefore, in order to update predictions after each time where subject i is observed, one must refit the model using a new risk set. The longer subject i is observed, the fewer individuals remain in the risk set and less data is available to do this.

### 3.4. Delayed kernel (DK) approach

We now introduce our DK approach to dynamic prediction. It aims to overcome some of the limitations of the standard JM and landmarking methods. Unlike landmarking, the DK approach aims to incorporate the entire data set, including the full history of covariate values while, at the same time remaining conceptually and computationally simpler than joint models.

#### 3.4.1. General setup

The starting point for the DK approach is an expression for the hazard rate that resembles that of weighted cumulative exposure models,^23,24^

(10) $$h^{\mathrm{DK}}(t|Z_{\left[0,s\right]})=h_{0}(t)\exp\;\left\{\int_{0}^{\min(s,t)}\sum_{\mu =1}^{p}\beta_{\mu }(t,t^{\prime },s)z_{\mu }(t^{\prime })dt^{\prime }\right\}$$

In this expression, the $\left\{z_{\mu }(t^{\prime })\right\}$ are time-dependent covariates, which we assume to be known from time 0 up to time s . To keep the notation compact we have left out time-independent covariates as these can always be included trivially with the addition of the term $\sum_{\nu =1}^{q}\gamma_{\nu }\zeta_{\nu }$ in the exponent. This model differs from the joint model approach to WCE in how we deal with covariates that are only observed up to some final observation time s before the event time. When t≤s (i.e. when t is a point in time prior to the last observation of covariates) the hazard rate in equation (10) only depends on covariates up to time t . For times t≥s covariates up to time s enter into the hazard rate.

The assumptions we make in writing expression (10) for the hazard function at time t , conditioned on covariate measurements recorded up to time s , are (a) proportional hazards (the logarithm of the hazard function depends linearly on the covariate trajectories), and (b) causality. If s<t we are not allowed to integrate over covariate values at times between s and t (since those are not conditioning variables). If s>t , causality prohibits including covariate values beyond time t (future covariate variations cannot influence the instantaneous hazard function). In combination this implies that the hazard function at time t has to involve integration over covariates up to time min(s,t) .

#### 3.4.2. Comparison between DK and existing models

In our view, there is no direct link between our present model (DK) and existing models such as JM, landmarking, or the more recent ‘landmarking 2.0’ (see Putter and van Houwelingen^40^ for details of this model); the latter follow different strategies. In JM one models the dynamics of the covariates in order to predict (probabilistically) their values at times beyond s . These predictions are then used in instantaneous Cox-type models. In landmarking the covariate history up until time point s is summarized in a set of sensible scalar covariates (i.e. covariates that do not depend on time, and are expected to capture the most relevant information that may influence future risk), which are then used as static covariates in a standard Cox model that interprets time s as the baseline. Landmarking 2.0^40^ is in fact closer to JM than to the original landmarking model, since (as in JM) it models the evolution of the covariates to predict values at times beyond s . It differs from standard JM in that the covariate predictions are subsequently used in a landmarking model rather than in a time-dependent Cox model.

In an early WCE model from Abrahamowicz et al.,^41^ the past covariate values are summarized in a time-dependent cumulative dose, which at time t is defined as a weighted average that integrates over all times up to t (in contrast to DK, which assumes that covariate data beyond time s are not available). In a paper by Sweeting et al.^42^ (model III) one finds a similar quantity, but now composed of covariate values at discrete past time points. The latter approach is most similar to our present model; however, in DK we do not write ad-hoc forms for the weighting function (the analogue of our kernel), but we seek to build suitable and more general parameterizations on the basis of plausible assumptions. Similarly, in models presented under the label ‘partly conditional modelling’^43^ one again builds on the instantaneous Cox model (see e.g. the first equation in Section 2.2 of Zheng and Heagerty,^43^ which the authors call their ‘general form’, and where the hazard function at time $s_{ik}$ depends only on longitudinal covariate values measured at time $s_{ik}$ ).

In their simplest form, all existing models build on the assumption that the instantaneous Cox model would hold if all covariate values at all times were known in full. They differ in how, from this starting point, the non-availability of covariate information in the time interval [s,t] is handled subsequently. In contrast, DK does not start from the assumption that the instantaneous Cox model would hold if covariate values at all times were known. This may or may not be the case, but is irrelevant in DK since it proceeds directly to parameterization of the hazard function, conditioned on knowledge of covariate values up to time s , and uses (what we believe to be natural and plausible) arguments for constraining the form of this parameterization: proportional hazards and causality.

**Figure 4.**
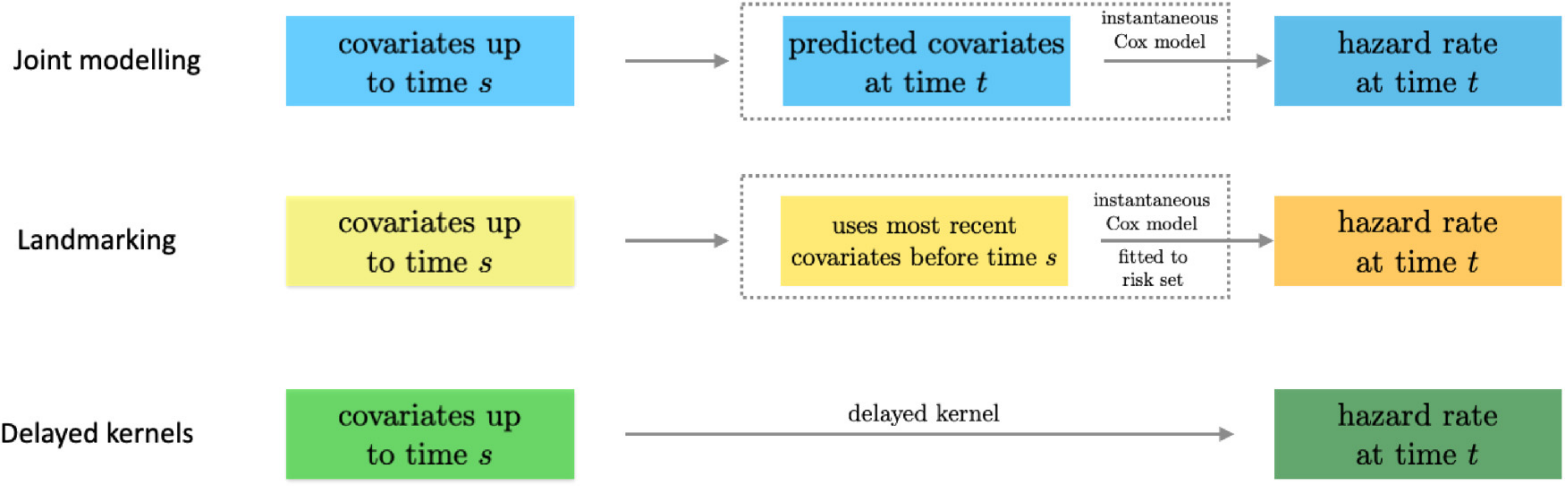
Relation of the delayed kernel approach to joint modelling and landmarking. All three approaches use covariates up to time s to make a prediction for an individual’s hazard rate at t . For the purposes of this figure, we assume t>s . Joint modelling first predicts future covariates (for times larger than s ), and then uses these predicted covariates at time t together with an instantaneous Cox-type model to construct a hazard rate for time t . Landmarking does not extrapolate or predict covariates for times later than s . Instead, it uses the last known covariates before time s as an input for a suitably constructed instantaneous Cox model (i.e. fitted to individuals still at risk of experiencing the event), which is then again used to generate a hazard rate at time t . In effect, both joint modelling and landmarking produce a predicted hazard rate at time t from the input of covariates at times up to s . This is done via the intermediate steps indicated by the dashed boxes in the figure. In the delayed kernel approach we do not construct any detailed mechanics inside those boxes. Instead, we directly parameterize the hazard rate at time t in terms of the covariate trajectory up to time s , see equation (10). It is important to note that equation (10) is not simply a representation of the physical or physiological effect of the covariate at an earlier time $t^{\prime }$ on the hazard rate at the later time t . Instead, the role of the delayed kernels is to statistically condition the hazard rate on the available information.

The relation between the three approaches is further summarized in the schematic diagram in Figure 4.

#### 3.4.3. Kernel definitions

The kernel $\beta_{\mu }(t,t^{\prime },s)$ describes (potentially) delayed effects of covariates. More precisely, $\beta_{\mu }(t,t^{\prime },s)$ quantifies the effect of the value of covariate μ at time $t^{\prime }$ on the hazard rate at a later time t , for a patient whose covariates are known up to time s . The form of equation (10) ensures causality, since only covariate values at times $t^{\prime }\leq t$ contribute to the hazard at time t . We set $\beta (t,t^{\prime },s)=0$ for $t^{\prime }>t$ . In principle, the precise form of $\beta_{\mu }(t,t^{\prime },s)$ could be chosen from a wide range of functions. We reduce this freedom via the following requirements which must hold for all μ :

i *Exponential decay of covariate impact.* We assume that the impact of each covariate μ at time $t^{\prime }$ on the hazard rate at a later time $t>t^{\prime }$ decays exponentially with the time difference $t-t^{\prime }$ . How fast the effect of the covariate decays is governed by a covariate-specific impact time scale $\tau_{\mu }\geq 0$ .
ii *Equivalence with standard Cox model for stationary covariates.* Our second requirement is that expression (10) reduces to the standard Cox model in equation (1) in the case of a constant covariate, that is, when $z_{\mu }(t)\equiv z_{\mu }$ for all t . This is achieved when there is a constant $a_{\mu }$ , which is independent of t and s , such that
(11) $$\int_{0}^{\min(s,t)}\beta_{\mu }(t,t^{\prime },s)dt^{\prime }=a_{\mu }$$
iii *Equivalence with instantaneous Cox model for short impact time scales.* Finally, for 0<t≤s we require that expression (10) reduces to the instantaneous Cox model in equation (3) in the limit $\tau_{\mu }↓0$ , that is, when the covariate impact on risk decays immediately. This is achieved, without violating (ii), if we have
(12) $$\lim_{\tau_{\mu }↓0}\beta_{\mu }(t,t^{\prime },s)=a_{\mu }\delta (t-t^{\prime })$$

From (i), it follows that our kernel $\beta_{\mu }(t,t^{\prime },s)$ must have the following form:

(13) $$\beta_{\mu }(t,t^{\prime },s)=A_{\mu }(t,s)\tau_{\mu }^{-1}e^{-(t-t^{\prime })/\tau_{\mu }}+B_{\mu }(t,s)$$

where the quantities $A_{\mu }(t,s)$ and $B_{\mu }(t,s)$ can depend on $\tau_{\mu }$ in general. Requirements (ii) and (iii) then translate into, respectively,

(14) $$\begin{array}{rl} s,t & \geq 0:\;A_{\mu }(t,s)e^{-t/\tau_{\mu }}(e^{\min(s,t)/\tau_{\mu }}-1)+\min(s,t)B_{\mu }(t,s)=a_{\mu } \end{array}$$

(15) $$\begin{array}{rl} 0<t & \leq s:\;\lim_{\tau_{\mu }↓0}A_{\mu }(t,s)=a_{\mu },\;\lim_{\tau_{\mu }↓0}B_{\mu }(t,s)=0 \end{array}$$

#### 3.4.4. Two models within this family

Finally, from the remaining family of models (those that satisfy equations (14) and (15)), we choose the two simplest members. These are defined by demanding that either $B_{\mu }(t,s)=0$ for *any* $\tau_{\mu }$ (model A), or that $A_{\mu }(t,s)=a_{\mu }$ for *any* $\tau_{\mu }$ (model B). Working out the details for these choices via equations (14) and (15) then leads to the following formulae:

(16) $$\begin{array}{rl} \text{Model A:}\;\beta_{\mu }^{A}(t,t^{\prime },s) & =\frac{a_{\mu }}{\tau_{\mu }}\frac{e^{t^{\prime }/\tau_{\mu }}}{e^{\min(s,t)/\tau_{\mu }}-1} \end{array}$$

(17) $$\begin{array}{rl} \text{Model B:}\;\beta_{\mu }^{B}(t,t^{\prime },s) & =\frac{a_{\mu }}{\tau_{\mu }}e^{-(t-t^{\prime })/\tau_{\mu }}+\frac{a_{\mu }}{\min(s,t)}\{1-e^{-t/\tau_{\mu }}(e^{\min(s,t)/\tau_{\mu }}-1)\} \end{array}$$

Both models are built around the time-translation invariant factor $\exp\;[-(t-t^{\prime })/\tau_{\mu }]$ and satisfy conditions (i), (ii), and (iii). So both reproduce the standard Cox model for time-independent covariates, as well as the instantaneous Cox model for longitudinal covariates with vanishing impact time scales, but they achieve this in distinct ways. We could have ensured a time-translation invariant kernel $\beta_{\mu }(t,t^{\prime },s)$ by choosing in equation (13) expressions for $A_{\mu }(t,s)$ and $B_{\mu }(t,s)$ that are independent of t . However, our models would then not reduce to the standard Cox model when covariates are constant. For t>s we find that $\beta_{\mu }^{A}(t,t^{\prime },s)$ is independent of t . This describes an anomalous response: the system ‘remembers’ early changes in covariates without decay. This could describe, for example, irreversible damage to the organism. In contrast, $\beta_{\mu }^{B}(t,t^{\prime },s)$ retains a decaying dependence on t when t>s , with $\lim_{t\to \infty }\beta_{\mu }^{B}(t,t^{\prime },s)=a_{\mu }/s$ . This could describe, for example, fluctuations in hormone levels that impact the hazard mostly in the short term, but also with persistent long-term effects. To aid the interpretation of the kernel functions we provide visualizations of kernels A and B in Section S1 of the Supplemental Material. Here, we plot equations (16) and (17) for a range parameter values and for the estimated parameters obtained from fitting the models to the Liver data set.

Equations (16) and (17) only hold for s>0 . In the data sets we study below, there are some individuals whose longitudinal covariates are observed only once at the baseline time (i.e. their final observation time is s=0 ). Given that equations (16) and (17) cannot be used for such individuals, we must specify their association parameters $\beta_{\mu }(t)$ in some other way. Two possible options are a constant association, $\beta_{\mu }(t)=a_{\mu }$ , or a decaying association, $\beta_{\mu }(t)=a_{\mu }e^{-t/\tau_{\mu }}$ . Throughout the main article, we choose the former in the DK models. Results for the decaying association are presented in Section S7 of the Supplemental Material.

We note that we condition on knowledge of the covariates observed over a specific time interval [0,s] in the model setup. As a consequence, the parameters in the DK models cannot necessarily be interpreted directly in terms of biophysical mechanisms. For example, $\tau_{\mu }$ encapsulates both the possible decay in the physical effect of covariate μ , and the decay that occurs from conditioning on knowledge of the covariate in the past. Parameter interpretations for the model, therefore, only make sense in a prediction context. We illustrate this point in a simple simulation study described briefly in Section 3.5 and in the Supplemental Material.

#### 3.4.5. Maximum likelihood inference

As in the standard Cox model, we use ML inference to determine the most plausible values of the model parameters based on the observed data. For simplicity, in this section, we will mostly omit the superscript DK from the hazard function. We write θ for the full set of parameters, that is, the model parameters $\left\{\tau_{\mu },a_{\mu }\right\}$ described in Section 3.4.4 and the baseline hazard function $h_{0}(t)$ , and assume initially that for each sample i the covariates are known over the full time interval $\left[0,s_{i}\right]$ . The optimal parameters are those for which the data likelihood P(D|θ) is maximized. For non-censored data, this likelihood is given by

(18) $$P(D|\theta )=\prod_{i=1}^{N}p(T_{i}|\theta ,Z_{\left[0,s_{i}\right]}^{i})$$

where $p(t|\theta ,Z_{\left[0,s_{i}\right]}^{i})$ is the probability density for individual i experiencing an event at time t given their covariate measurements. This probability density is expressed in terms of the parameterized hazard rate $h(t|\theta ,Z_{\left[0,s_{i}\right]}^{i})$ and the survival probability $S(t|\theta ,Z_{\left[0,s_{i}\right]}^{i})=\exp\;[-\int_{0}^{t}dt^{\prime }h(t^{\prime }|\theta ,Z_{\left[0,s_{i}\right]}^{i})]$ via

(19) $$p(t|\theta ,Z_{\left[0,s_{i}\right]}^{i})=h(t|\theta ,Z_{\left[0,s_{i}\right]}^{i})S(t|\theta ,Z_{\left[0,s_{i}\right]}^{i})$$

For right-censored data, there are two contributions to the likelihood. Individuals for whom an event is observed at time $T_{i}=T_{i}^{*}$ contribute a density $p(T_{i}|\theta ,Z_{\left[0,s_{i}\right]}^{i})$ . Those that are censored at time $T_{i}=C_{i}$ contribute the survival probability $S(T_{i}|\theta ,Z_{\left[0,s_{i}\right]}^{i})$ . Using the primary event indicator $\delta_{i}=I(T_{i}^{*}\leq C_{i})\in \{0,1\}$ , the likelihood for censored data is then

(20) $$P(D|\theta )=\prod_{i=1}^{N}h(T_{i}|\theta ,Z_{\left[0,s_{i}\right]}^{i}{)}^{\delta_{i}}S(T_{i}|\theta ,Z_{\left[0,s_{i}\right]}^{i})$$

Upon defining $\Omega_{\mathrm{ML}}(\theta )=-\log\;P(D|\theta )$ , we can write the ML parameter estimators as ${\hat{\theta }}_{\text{ML}}={\text{argmin}}_{\theta }\Omega_{\text{ML}}(\theta )$ .

A full derivation of the ML equations for models of the form in equation (10) is provided in Section S2.1 of the Supplemental Material. Here we present only the results. The ML estimator of the baseline hazard function is the direct analogue of the Breslow estimator,^39^

(21) $$\begin{array}{r} {\hat{h}}_{0}(t)=\frac{\sum_{i=1}^{N}\delta_{i}\delta (t-T_{i})}{\sum_{i=1}^{N}I(t\in [0,T_{i}])e^{\sum_{\mu }\int_{0}^{\min(s_{i},t)}\beta_{\mu }(t,t^{\prime },s_{i})z_{\mu }^{i}(t^{\prime })dt^{\prime }}} \end{array}$$

recalling from Section 3.1 that I(A)=1 if A holds, and I(A)=0 otherwise. The remaining parameters $\left\{a_{\mu },\tau_{\mu }\right\}$ in equations (16) and (17) are found by minimization of

(22) $$\begin{array}{rl} \Omega_{\mathrm{ML}}[\{a_{\mu },\tau_{\mu }\}] & =\sum_{i=1}^{N}\delta_{i}\log\;(\sum_{j=1}^{N}I(T_{i}\in [0,T_{j}])e^{\sum_{\mu }\int_{0}^{\min(s_{j},T_{i})}\beta_{\mu }(T_{i},t^{\prime },s_{j})z_{\mu }^{j}(t^{\prime })dt^{\prime }})\\ & \;-\sum_{i=1}^{N}\delta_{i}\sum_{\mu }\int_{0}^{s_{i}}\beta_{\mu }(T_{i},t^{\prime },s_{i})z_{\mu }^{i}(t^{\prime })dt^{\prime } \end{array}$$

where we have disregarded terms that are independent of $\left\{a_{\mu },\tau_{\mu }\right\}$ . As in all Cox-type models, the final minimization of equation (22) with respect to the remaining parameters (here, the associations and time scales) must be performed numerically, for example, using Powell’s method.^44^

Once we have obtained the ML estimates of the model parameters $\left\{a_{\mu },\tau_{\mu }\right\}$ , we substitute these values into equation (16) or (17) as appropriate to obtain an estimate of the kernel function $\beta (t,t^{\prime },s)$ . This kernel is then inserted, along with the covariate trajectory, into equations (21) and (10) to estimate the hazard function. For simple covariate interpolation procedures (see Section 3.4.7) the integrals in the hazard function can be evaluated analytically. In Section S2.4 of the Supplemental Material we evaluate these expressions explicitly.

#### 3.4.6. Dynamic prediction

Using the ML estimates ${\hat{\theta }}_{\mathrm{ML}}$ for the model parameters, we can use the DK models to estimate the quantity $\pi (u|Z_{\left[0,s\right]},s)$ in equation (4), representing the probability that a subject has not experienced an event by time u>s , conditional on their survival to s and on their covariate values $Z_{\left[0,s\right]}$ up to that time. That is

(23) $${\hat{\pi }}^{\mathrm{DK}}(u|Z_{\left[0,s\right]},s)=\exp\;\{-\int_{s}^{u}{\hat{h}}^{\mathrm{DK}}(t^{\prime }|Z_{\left[0,s\right]})dt^{\prime }\}$$

with ${\hat{h}}^{\mathrm{DK}}(t|Z_{\left[0,s\right]})$ as defined by equation (10), with kernels of the form in equations (16, 17) and with the ML estimators for the parameters in those kernels. We note that equation (23) gives simply the relation between survival probability and hazard rates; it does not imply any further assumptions beyond those underlying the parameterization chosen for the hazard rate in equation (10) (viz. proportional hazards and causality). Using the ML estimator in equation (21) of the baseline hazard function we can perform the integration in equation (23) to find

(24) $${\hat{\pi }}^{\mathrm{DK}}(u|Z_{\left[0,s\right]},s)=\exp\;\{-\sum_{j=1}^{N}\delta_{j}\;I(T_{j}\in [s,u])\frac{e^{\sum_{\mu =1}^{p}\int_{0}^{s}{\hat{\beta }}_{\mu }(T_{j},t^{\prime },s)z_{\mu }(t^{\prime })dt^{\prime }}}{\sum_{k=1}^{N}I(T_{j}\in [0,T_{k}])e^{\sum_{\mu }\int_{0}^{\min(s_{k},T_{j})}{\hat{\beta }}_{\mu }(T_{j},t^{\prime },s_{k})z_{\mu }^{k}(t^{\prime })dt^{\prime }}}\}$$

where ${\hat{\beta }}_{\mu }(t,t^{\prime },s)$ indicates the association kernel obtained from the ML estimators of the parameters $\left\{a_{\mu },\tau_{\mu }\right\}$ . In the numerator, we have used the fact that the prefactor $I(T_{j}\in [s,u])$ ensures that $\min(s,T_{j})=s$ . In equation (24) the j and k indices label the individuals in the data set used for inference. Variables without index labels (e.g. s , $Z_{\left[0,s\right]}$ , $z_{\mu }(t)$ ) refer to the individual (not in the data set) for whom we are making predictions.

Any uncertainty in the prediction (23) reflects uncertainty in the inferred model parameters. It is hence quantified by the variance of the covariate-conditioned survival function, calculated with respect to the posterior parameter distribution (given the data). The parameter distribution is approximated by a Gaussian distribution (for sufficiently large sample sizes), centered at the ML point, and with a covariance matrix obtained via the Fisher information.

#### 3.4.7. Covariate interpolation

So far, we have defined the DK models conditional on covariate trajectories $Z_{\left[0,s\right]}$ over the entire interval [0,s] . In reality, we do not have full knowledge of these trajectories. Instead for each subject i we have a finite number of discrete measurements that coincide with follow-up appointments, $Z^{i}=\{z_{\mu }^{i}(t_{i\ell });\;\mu =1,\ldots ,p,\ell =1,\ldots ,n_{i},t_{i\ell }\in [0,s_{i}]\}$ . In order to perform the integrals in equations (10), (22) and (24) we must interpolate between these discrete observed values.

We choose the simple “last observation carried forward” (LOCF) procedure, commonly used in instantaneous Cox models and landmarking approaches. That is, $z_{\mu }^{i}(t)=z_{\mu }^{i}(t_{i\ell })$ where $t_{i\ell }$ is the latest observation time before (or equal to) t , that is, $\max\{t_{i\ell }:t_{i\ell }\leq t\}$ . Using this method, the integrals in equations (10), (22) and (24) can be evaluated analytically (see Section S2.3 of the Supplemental Material). This reduces the computational effort required to perform the minimization and the dynamic prediction. Other, smoother interpolation procedures such as Gaussian convolutions^45,46^ are also possible and may improve estimations (at some computational cost). While interpolation makes assumptions about the values of the covariate within the observation interval $\left[0,s_{i}\right]$ , we do not make assumptions about the covariates after the final observation time $s_{i}$ .

### 3.5. Simulation study

To understand better the DK model and how to interpret the associated parameters, we perform a simple simulation study. Generating data according to the DK model is non-trivial since event times are dependent on the period over which the covariates are observed. Instead, we generate data according to a simple joint model (with longitudinal measurements generated from a linear random slope and random intercept model) and compare parameter estimates between the models. We do this for a joint model with an instantaneous association function in the survival sub-model (scenario 1) and for a joint model with a cumulative association (scenario 2). In each scenario we fit both DK models, a landmarking model and two joint models (one with an instantaneous survival model and one with a cumulative survival model). We repeat the simulation 50 times and inspect the average behavior of the different models.

Details of the methodology and results of the simulation are provided in Section S3 of the Supplemental Material. We briefly describe the main findings here. First, we find that the fixed associated parameter $\gamma_{\nu }$ is well estimated by the joint model that generated the data, but slightly underestimated when the survival sub-model is mis-specified. The estimate of the equivalent parameter in the DK models is comparable but smaller than the value in the joint models. This suggests a similar but not identical interpretation of this parameter in the DK and joint model frameworks. In scenario 1 (instantaneous association), parameter $a_{\mu }$ in DK models A and B is, on average, estimated to be similar to the association parameter $\alpha_{\mu }$ in the correctly specified joint model. The joint model with a cumulative association (mis-specified) typically underestimates this parameter. In scenario 2 (cumulative association), the cumulative joint model correctly estimates $\alpha_{\mu }$ while the instantaneous joint model overestimates on average. The parameter $a_{\mu }$ in the DK model is consistently estimated to be in the same direction as $\alpha_{\mu }$ (indicating the same direction of covariate effect) but is larger in magnitude. Parameter $a_{\mu }$ is a parameter of the delayed kernels which, according to equations (16) and (17), controls the overall impact of conditioning covariate μ on the hazard (independent of specific temporal aspects). It has a similar but distinct interpretation to $\alpha_{\mu }$ in the JM framework which, for an instantaneous model (scenario 1), is the association between the hazard at time t and the predicted longitudinal variable at that time. For the cumulative JM model (scenario 2), $\alpha_{\mu }$ is the association between the hazard at time t and the predicted longitudinal trajectory integrated from baseline up to t .

Finally, we find that even when events are generated from a survival sub-model with an instantaneous association, the DK models estimate a non-zero decay parameter, τ>0 . This demonstrates our earlier assertion that $\tau_{\mu }$ does not simply represent the decay in the physical effect of covariate μ . Instead, the interaction kernels of models A and B mediate statistical conditioning information, not necessarily direct mechanical influence. Since the DK model gives event time statistics conditioned on the past, as soon as covariate information is unavailable in between s and t , the event probability density at time t conditioned on covariates up to time s must represent the result of marginalization over covariate values in the interval [s,t] . Such marginalization will lead to a dependence on past covariate values that is no longer local in time. Thus, past covariate values may influence current hazards in a situation when only present values have a mechanical impact on risk, simply because the past values are correlated with the current covariate values.

In a couple of iterations of the simulation, the DK models estimated values of $a_{\mu }$ and $\gamma_{\mu }$ that were very different from their typical values. This could be due to an issue with the implementation of these models, for example, in finding the global maximum of the likelihood function. The estimated value of the decay parameter, $\tau_{\mu }$ , was unstable with many iterations estimating extreme values. More extensive simulations, including those with data generated from retarded kernel models, are required to investigate this behavior further.

## 4. Application to clinical data

### 4.1. Methods

#### 4.1.1. Cross-validation

For each of the data sets in Section 2, we assess the predictive accuracy of the different dynamic prediction models using 10-fold cross-validation. The data is split randomly into 10 equally sized groups. At each iteration, one group is assigned as the test data set and the remaining groups make up the training data set. Each model is fitted to the training data and the resulting model is used to make survival predictions about individuals in the test data set. Predictive accuracy is assessed by comparing these predictions to the true event times in the test data (see Section 4.1.3). The procedure was repeated 10 times with each group being assigned as the test data.

#### 4.1.2. Fitting models

We implemented the DK models using our own Python code which performs the ML estimation using Powell minimization.^44^ Joint models were fitted using the JMbayes2 package and landmarking models were fitted using the standard survival package in R. Details of the joint model implementation including the convergence settings used are provided in Section S4 of the Supplemental Material.

#### 4.1.3. Measuring predictive accuracy

Following Rizopoulos et al.,^2^ we quantify the predictive accuracy of the different models using the expected error of predicting future events. Dynamic prediction is concerned with predicting the survival of individuals to a given time u , based on their survival to some earlier time t<u , and covariate measurements for the individual up to this time. The expected prediction error for a given ‘prediction time’ u and ‘base time’ t is then defined as follows^47^:

(25) $$\mathrm{PE}(u|t)=E\left[L\{N_{i}(u)-\pi (u|Z_{\left[0,t\right]}^{i},t)\}\right]$$

where $N_{i}(u)=I(T_{i}^{*}>u)$ is the true event status of subject i at time u , and $\pi (u|Z_{\left[0,t\right]}^{i},t)$ is the model’s predicted survival probability for subject i based on information about this subject (covariate measurements and survival status) up to the base time t . The notation E stands for an average over the distribution of covariates and event times. L(.) denotes a loss function which defines how we measure the difference between survival status and predicted survival probability. Common choices are $L(x-\hat{x})=|x-\hat{x}|$ and the squared loss $L(x-\hat{x})=(x-\hat{x}{)}^{2}$ (where x is the target parameter and $\hat{x}$ is its predicted value).^2,32,47^ We choose the latter. The definition of prediction error is such that PE(u|t)=0 if the survival status of all individuals is predicted with full accuracy (i.e. $\pi (u|Z_{\left[0,t\right]}^{i},t)=1$ for all subjects who are alive at time u and $\pi (u|Z_{\left[0,t\right]}^{i},t)=0$ for subjects who are dead by time u ). If the reverse is true ( $\pi (u|Z_{\left[0,t\right]}^{i},t)=1$ for subjects who are dead at time u and $\pi (u|Z_{\left[0,t\right]}^{i},t)=0$ for subjects who are alive) then PE(u|t)=1 . We obtain PE(u|t)=0.25 if every individual has predicted survival probability $\pi (u|Z_{\left[0,t\right]}^{i},t)=0.5$ .

Again following Rizopoulos et al.,^2^ in this article, we use the overall prediction error PE(u|t) proposed by Henderson et al.,^32^ that in addition takes into account censoring

(26) $$\begin{array}{rl} \hat{\mathrm{PE}}(u|t) & =\frac{1}{n(t)}\sum_{i;T_{i}\geq t}I(T_{i}\geq u)L\{1-\hat{\pi }(u|Z_{\left[0,t\right]}^{i},t)\}+\delta_{i}I(T_{i}<u)L\{0-\hat{\pi }(u|Z_{\left[0,t\right]}^{i},t)\}\\ & \;+(1-\delta_{i})I(T_{i}<u)[\hat{\pi }(u|Z_{\left[0,t\right]}^{i},T_{i})L\{1-\hat{\pi }(u|Z_{\left[0,t\right]}^{i},t)\}+\{1-\hat{\pi }(u|Z_{\left[0,t\right]}^{i},T_{i})\}L\{0-\hat{\pi }(u|Z_{\left[0,t\right]}^{i},t)\}] \end{array}$$

The sum extends over the n(t) subjects in the test data set who are still at risk at time t . The first term of equation (26) corresponds to individuals in the test data who are still alive after time u . These have survival status $N_{i}(u)=1$ , and, therefore, contribute a loss function based on the difference between their estimated survival probability and 1, that is, $L\{1-\hat{\pi }(u|Z_{\left[0,t\right]}^{i},t)\}$ . The second term refers to individuals who have experienced an event by time u (i.e. $T_{i}=T_{i}^{*}<u$ ). Their survival status is 0 and therefore they contribute a loss function $L\{0-\hat{\pi }(u|Z_{\left[0,t\right]}^{i},t)\}$ . The final term represents individuals who were censored before time u (i.e. $T_{i}=C_{i}\in [t,u]$ ) so we do not know their survival status at time u . Here the estimated probability of survival based on information up to time t is compared with the probability of survival given that we know subject i survived up until their censoring time $T_{i}\geq t$ .

To compare the predictive accuracy of JM, landmarking and the DK approach we insert into equation (26) the respective estimators ${\hat{\pi }}^{\mathrm{JM}}(u|Z_{\left[0,t\right]}^{i},t)$ , ${\hat{\pi }}^{\mathrm{LM}}(u|Z_{\left[0,t\right]}^{i},t)$ and ${\hat{\pi }}^{\mathrm{DK}}(u|Z_{\left[0,t\right]}^{i},t)$ . This requires that we calculate the probability of a subject’s survival to time u , based on survival and covariate observations until a general base time t<u that need not be the individual’s final observation time $s_{i}$ . For the joint model and landmarking estimators we replace the final observation time with t in equations (7) and (9). For the DK estimator ${\hat{\pi }}^{\mathrm{DK}}(u|Z_{\left[0,t\right]}^{i},t)$ in equation (24), we replace $I(T_{j}\in [s_{i},u])$ with $I(T_{j}\in [t,u])$ since we know subject i is alive until t . However, we only have covariate observations up to the latest observation time $t_{i\ell }$ that is ≤t . In line with our chosen interpolation procedure, we only integrate the covariate trajectory up to this time. Specifically, for any general base time t we have

(27) $${\hat{\pi }}^{\mathrm{DK}}(u|Z_{\left[0,t\right]}^{i},t)=\exp\;\left\{-\sum_{j=1}^{N}\delta_{j}\;I(T_{j}\in [t,u])\frac{e^{\sum_{\mu }\int_{0}^{\max\{t_{i\ell }:t_{i\ell }\leq t\}}{\hat{\beta }}_{\mu }(T_{j},t^{\prime },\max\{t_{i\ell }:t_{i\ell }\leq t\})z_{\mu }^{i}(t^{\prime })dt^{\prime }}}{\sum_{k=1}^{N}I(T_{j}\in [0,T_{k}])e^{\sum_{\mu }\int_{0}^{\min(s_{k},T_{j})}{\hat{\beta }}_{\mu }(T_{j},t^{\prime },s_{k})z_{\mu }^{k}(t^{\prime })dt^{\prime }}}\right\}$$

where index i labels the individual (in the test data) for whom we are making predictions, while the sums over j and k refer to individuals in the training data set used for inference. The integral limit $\max\{t_{i\ell }:t_{i\ell }\leq t\}$ labels the last observation time of individual i before (or at) the base time t .

The term $\hat{\pi }(u|Z_{\left[0,t\right]}^{i},T_{i})$ in equation (26) represents the probability of survival to u given subject i survived to their censoring time $T_{i}=C_{i}$ . To calculate this using the DK model, we replace $I(T_{j}\in [t,u])$ with $I(T_{j}\in [T_{i},u])$ in equation (27). For JM, ${\hat{\pi }}^{\mathrm{JM}}(u|Z_{\left[0,t\right]}^{i},T_{i})$ is obtained by replacing $Z_{\left[0,s\right]}$ with $Z_{\left[0,t\right]}^{i}$ and by replacing the condition $T^{*}>s$ with $T_{i}^{*}>T_{i}$ in equation (7). Since this term is only calculated for censored individuals ( $T_{i}=C_{i}$ ), the condition $T_{i}^{*}>T_{i}$ means “the true event time of individual i is greater than their censoring time.” Finally, for landmarking we use ${\hat{\pi }}^{\mathrm{LM}}(u|Z_{\left[0,t\right]}^{i},T_{i})={\hat{\pi }}^{\mathrm{LM}}(u|Z_{\left[0,t\right]}^{i},t)/{\hat{\pi }}^{\mathrm{LM}}(T_{i}|Z_{\left[0,t\right]}^{i},t)$ which is equivalent to replacing s with t in equation (9) except in the integral limits where we replace $\int_{s}^{u}$ with $\int_{T_{i}}^{u}$ .

To perform the prediction error calculation for the DK models, we use our own Python code following equations (26) and (27). For joint models, we use the tvBrier function in the JMbayes2 package and for landmarking we use the Cox model version of prederrJM in JMbayes. To ensure consistency between all prediction error evaluations we make minor modifications to the tvBrier and prederrJM functions (see Section S6 of the Supplemental Material for details).

#### 4.1.4. Fixed base time

First, we compare the predictive accuracy of the three methods by specifying a fixed base time t and varying the prediction time u . Based on Figures 1 and 3, for the PBC and Liver data sets we choose a fixed base time of t=3 years. This value is chosen so that a large number of individuals are still alive after this time (and we can hence make predictions about them), but also so that these individuals have had their covariates measured multiple times before this time. We then vary the prediction time u from the base time t=3 years in steps of 0.2 years up to 8 years for the PBC data, and up to 10 years for the Liver data. For the AIDS data set, we choose t=6 months as the base time, so that most individuals have been observed three times. We then vary the prediction time u from this base time up to 18 months in steps of 0.2 months.

#### 4.1.5. Fixed prediction window

In our second test, we vary the base time t , while keeping the prediction window w=u−t fixed (i.e. the time difference between prediction and base time). Since we are varying the base time t , we must then fit a new landmark model for each choice of t (where the landmark time υ=t ). On the other hand, for the DK approach and the joint model, we need only fit the model once and can make the error assessments at each iteration using this single fitted model.

Based on previous analysis of the PBC and Liver data,^16,32^ we choose three prediction windows: $w_{1}=1$ year, $w_{2}=2$ years, and $w_{3}=3$ years. Given the event time distributions, we do not make predictions for either data set beyond u=10 years. Therefore, for $w_{1}$ , we vary the base time from 0 to 9 years, for $w_{2}$ , we vary it from 0 to 8 years and for $w_{3}$ , this is 0–7 years. In all cases, we increase the base time in increments of 0.2 years.

Based on the event time distribution of the AIDS data, we choose prediction windows $w_{1}=6$ months, $w_{2}=9$ months and $w_{3}=12$ months. Here covariates are observed at 0, 2, 6, 12, and 18 months only. As a result, predictions will only be updated at these time steps, and we can only make a small number of distinct measurements of predictive accuracy. Due to the event times in the AIDS data set, we do not make predictions past 18 months. Therefore, for window $w_{1}$ , we use base times t=0,2,6,12 months and for windows $w_{2}$ and $w_{3}$ we use t=0,2,6 months only.

### 4.2. PBC data set

We fit each model to the PBC training data set using p=3 time-dependent covariates and a single fixed covariate: $z_{1}^{i}(t)$ denotes log serum bilirubin, $z_{2}^{i}(t)$ denotes log serum albumin, $z_{3}^{i}(t)$ is the log prothrombin time, and the fixed covariate $\zeta_{1}^{i}$ is the subject’s age at baseline.

The PBC data set contains event-time information for two events, death and liver transplant. The most appropriate way of analysing this data is to use a competing risks model. However, for simplicity, we here treat the transplant event as a censoring event. Another simple way to analyse this data is to treat the two events as a single composite event. We provide the results of the latter analysis in Section S5 of the Supplemental Material. The two analyses are found to give similar results.

#### 4.2.1. Models

For the joint model, we first fit a simple multivariate linear mixed model to each of the three time-dependent covariates

(28) $$z_{\mu }^{i}(t)=m_{\mu }^{i}(t)+\varepsilon_{\mu }^{i}(t)=\eta_{\mu ,0}+b_{\mu ,0}^{i}+(\eta_{\mu ,1}+b_{\mu ,1}^{i})t+\varepsilon_{\mu }^{i}(t)$$

where the random effects $b^{i}$ are assumed to follow a joint multivariate normal distribution with mean zero and variance-covariance matrix D .

Figure 1 suggests that the covariate trajectories in the PBC data may be non-linear for some individuals. Hence, for extra flexibility, we also fit a second joint model that includes natural cubic splines in both the fixed and random effects parts of the model. Following Rizopoulos,^25^ the log serum bilirubin ( μ=1 ) is modelled using natural cubic B-splines with 2 degrees of freedom

(29) $$\begin{array}{rl} z_{1}^{i}(t) & =m_{1}^{i}(t)+\varepsilon_{1}^{i}(t)\\ & =\eta_{1,0}+b_{1,0}^{i}+(\eta_{1,1}+b_{1,1}^{i})B_{1}^{n}(t,\lambda )+(\eta_{1,2}+b_{1,2}^{i})B_{2}^{n}(t,\lambda )+\varepsilon_{1}^{i}(t) \end{array}$$

where $\left\{B_{k}^{n}(t,\lambda );k=1,2\right\}$ denotes the B-spline basis matrix for a natural cubic spline of time.^16,48^ We write analogous equations for both the log albumin and the log prothrombin covariates. Again, the random effects of all three longitudinal covariates are assumed to follow a joint multivariate normal distribution.

For both the linear and spline longitudinal models, the hazard function of the survival sub-model in the JM framework is

(30) $$\begin{array}{r} h^{\mathrm{JM}}(t|M_{\left[0,t\right]}^{i})=h_{0}(t)\exp\;\left\{\gamma_{1}\zeta_{1}^{i}+\alpha_{1}m_{1}^{i}(t)+\alpha_{2}m_{2}^{i}(t)+\alpha_{3}m_{3}^{i}(t)\right\} \end{array}$$

where we recall from Section 3.2 that $M_{\left[0,t\right]}^{i}=\{m_{\mu }^{i}(t^{\prime });\;\mu =1,\ldots ,p,\;t^{\prime }\in [0,t]\}$ denotes the history of the ‘true’ (unobserved) longitudinal covariates up to time t for subject i . For the landmark model, the hazard is instead specified for a given landmark time, ,

(31) $$h^{\mathrm{LM}}(t|Z^{i},\upsilon )=h_{0}(t|\upsilon )\exp\;\left\{\gamma_{1}\zeta_{1}^{i}+\alpha_{1}(\upsilon ){\tilde{z}}_{1}^{i}(\upsilon )+\alpha_{2}(\upsilon ){\tilde{z}}_{2}^{i}(\upsilon )+\alpha_{3}(\upsilon ){\tilde{z}}_{3}^{i}(\upsilon )\right\}$$

where ${\tilde{z}}_{\mu }^{i}(\upsilon )$ is again the last observed value of covariate μ for patient i before time υ .

For the DK approach, we specify the hazard function as

(32) $$\begin{array}{rl} h^{\mathrm{DK}}(t|Z_{\left[0,s_{i}\right]}^{i}) & =h_{0}(t)\exp\;\{\gamma_{1}\zeta_{1}^{i}+\int_{0}^{\min(s_{i},t)}\left(\beta_{1}(t,t^{\prime },s_{i})z_{1}^{i}(t^{\prime })+\beta_{2}(t,t^{\prime },s_{i})z_{2}^{i}(t^{\prime }\right)\\ & \;+\;\beta_{3}(t,t^{\prime },s_{i})z_{3}^{i}(t^{\prime }))dt^{\prime }\} \end{array}$$

The parameterizations of the time-dependent association parameters $\beta_{\mu }(t,t^{\prime },s)$ are given in equations (16) and (17) for models A and B, respectively.

#### 4.2.2. Results

**Figure 5.**
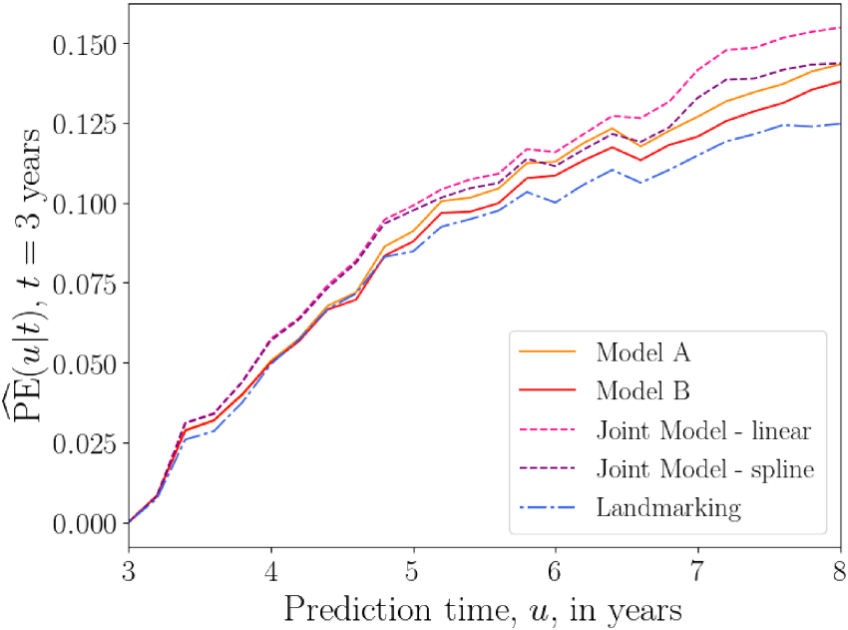
Overall prediction error $\hat{\mathrm{PE}}(u|t)$ as a function of prediction time u (in years) for the PBC data with fixed base time t=3 years. Prediction error is calculated for u values from 3 to 8 years, with 0.2 year increments. A squared loss function was used in equation (26). The prediction error plotted at each time u is an average over values of $\hat{\mathrm{PE}}(u|t)$ calculated at each iteration of the 10-fold cross-validation procedure. The results from models A and B of the delayed kernel approach are plotted alongside the landmarking model and two joint models (one that uses a linear longitudinal model for the time-dependent covariates, and another that uses cubic splines). PE: prediction error; PBC: primary biliary cirrhosis.

Figure 5 shows plots of the overall prediction error $\hat{\mathrm{PE}}(u|t)$ against the prediction time u for a fixed base time of t=3 years estimated via 10-fold cross-validation. Results for the linear joint model, spline joint model, landmarking model and models A and B of the DK approach are plotted on the same graph. Up to u=5 years, the DK models and landmarking model exhibit similar prediction errors, slightly outperforming the two joint models. At later prediction times, landmarking performs best followed by DK model B. The linear joint model has the highest prediction error.

**Figure 6.**
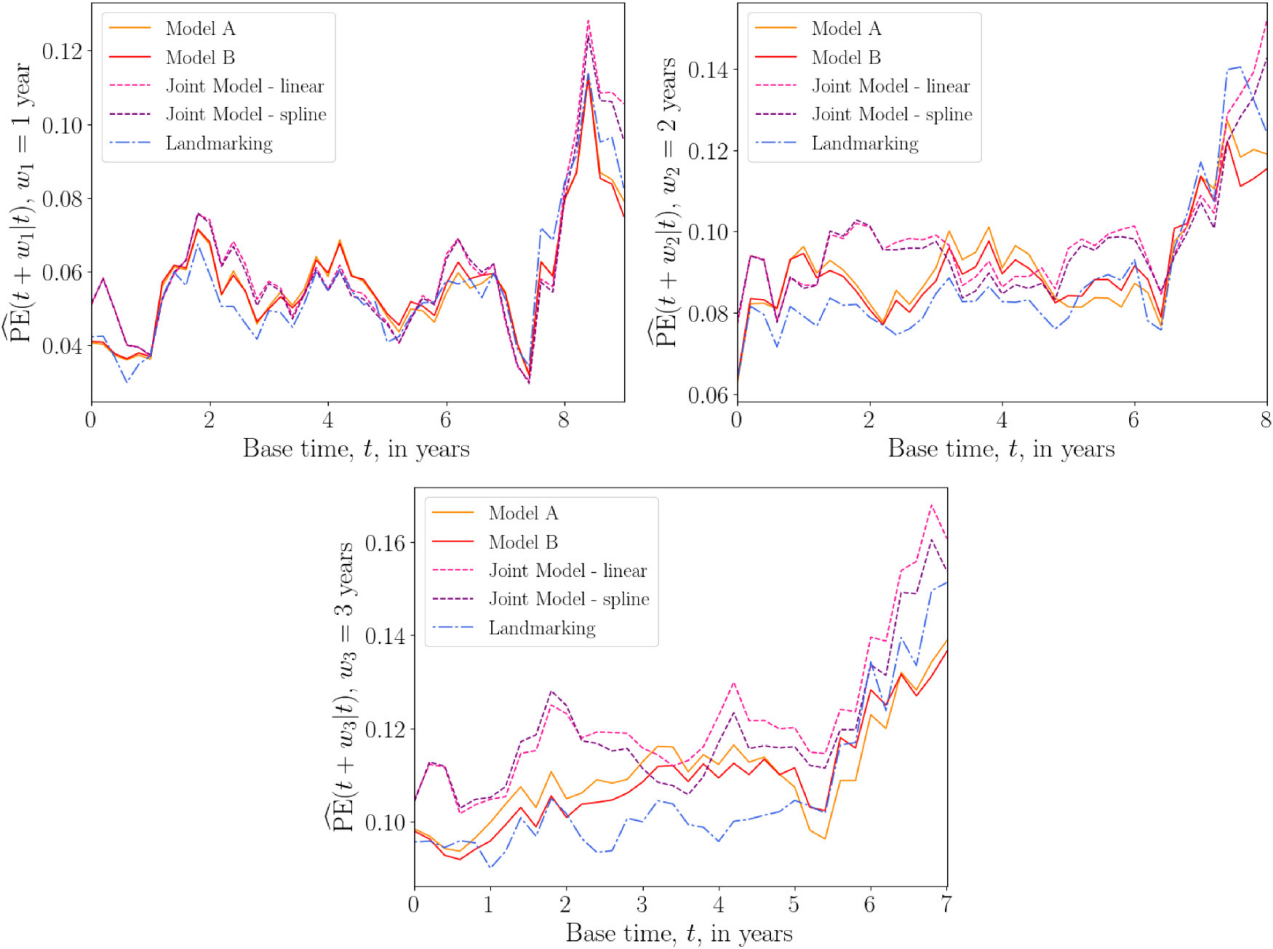
Overall prediction error $\hat{\mathrm{PE}}(u|t)$ versus base time t (in years) for the PBC data, with prediction windows $w_{1}=1$ year, $w_{2}=2$ years and $w_{3}=3$ years. The prediction times are u=t+w . The prediction error is calculated for t ranging from 0 to 9, 8 or 7 years for $w_{1}$ , $w_{2}$ and $w_{3}$ , respectively, with 0.2 year increments. A squared loss function was used in equation (26). The prediction error plotted at each time t is an average over values of $\hat{\mathrm{PE}}(u|t)$ calculated at each iteration of the 10-fold cross-validation procedure. Results from models A and B of the delayed kernel approach are plotted alongside the landmarking model and two joint models; one that uses a linear longitudinal model for the time-dependent covariates, and another that uses cubic splines. PE: prediction error; PBC: primary biliary cirrhosis.

Plots of the average prediction error $\hat{\mathrm{PE}}(u|t)$ against the base time t are shown in Figure 6, for fixed prediction windows $w_{1}=1$ year, $w_{2}=2$ years, and $w_{3}=3$ years. Again results for the five different models are plotted in the same graphs. For the shortest prediction window $w_{1}$ , all models are similarly accurate. For the larger prediction windows, the two joint models perform slightly worse on average compared with the other models. For prediction window $w_{3}=3$ , landmarking tends to have the lowest prediction error for base times up to t=5 years, after which the DK models start to perform best. The DK models outperform the others for all windows in the very final months of predictions.

The results of the above tests suggest that, for the PBC data, the DK approach performs as well as, and sometimes better than, existing methods. Care should be taken when interpreting these results, as we have not used a competing risks model in our analysis. This data set does, however, serve as an illustration that, without sacrificing accuracy, the DK model can serve as a simpler alternative to JM when considering multiple longitudinal covariates.

### 4.3. AIDS data set

In the AIDS data set, we focus on a single longitudinal covariate, the CD4 count $z_{1}^{i}(t)$ . We also include four fixed binary covariates: drug group ( $\zeta_{1}^{i}=1$ for ddI and $\zeta_{1}^{i}=0$ for ddC), gender ( $\zeta_{2}^{i}=1$ for male and $\zeta_{2}^{i}=0$ for female), PrevOI ( $\zeta_{3}^{i}=1$ for AIDS diagnosis at study entry and $\zeta_{3}^{i}=0$ for no AIDS diagnosis) and stratum ( $\zeta_{4}^{i}=1$ for AZT failure and $\zeta_{4}^{i}=0$ for AZT intolerance). See Section 2.2 for a description of these variables.

#### 4.3.1. Models

The JM framework allows us to model the dependence of CD4 count on the patients drug group. Following Rizopoulos,^16^ we fit the linear mixed model

(33) $$\begin{array}{rl} z_{1}^{i}(t) & =m_{1}^{i}(t)+\varepsilon_{1}^{i}(t)\\ & =\eta_{1,0}+b_{1,0}^{i}+(\eta_{1,1}+b_{1,1}^{i})t+\eta_{1,2}\zeta_{1}^{i}t+\varepsilon_{1}^{i}(t) \end{array}$$

where the term $\eta_{1,2}\zeta_{1}^{i}t$ denotes the effect of the interaction of treatment (drug group) with time. As usual, the random effects $b^{i}$ are assumed to follow a normal distribution. To complete the joint model, the hazard function is then chosen as

(34) $$h^{\mathrm{JM}}(t|M_{\left[0,t\right]}^{i})=h_{0}(t)\exp\;\left\{\gamma_{1}\zeta_{1}^{i}+\gamma_{2}\zeta_{2}^{i}+\gamma_{3}\zeta_{3}^{i}+\gamma_{4}\zeta_{4}^{i}+\alpha_{1}m_{1}^{i}(t)\right\}$$

For the landmark model with landmark time υ one has

(35) $$h^{\mathrm{LM}}(t|Z^{i},\upsilon )=h_{0}(t|\upsilon )\exp\;\left\{\gamma_{1}\zeta_{1}^{i}+\gamma_{2}\zeta_{2}^{i}+\gamma_{3}\zeta_{3}^{i}+\gamma_{4}\zeta_{4}^{i}+\alpha_{1}(\upsilon ){\tilde{z}}_{1}^{i}(\upsilon )\right\}$$

and for the DK approach we specify the survival model as follows:

(36) $$h^{\mathrm{DK}}(t|Z_{\left[0,s_{i}\right]}^{i})=h_{0}(t)\exp\;\{\gamma_{1}\zeta_{1}^{i}+\gamma_{2}\zeta_{2}^{i}+\gamma_{3}\zeta_{3}^{i}+\gamma_{4}\zeta_{4}^{i}+\int_{0}^{\min(s_{i},t)}\beta_{1}(t,t^{\prime },s_{i})z_{1}^{i}(t^{\prime })dt^{\prime }\}$$

As before, the parameterizations of $\beta_{\mu }(t,t^{\prime },s)$ in models A and B are given in equations (16) and (17), respectively.

#### 4.3.2. Results

**Figure 7.**
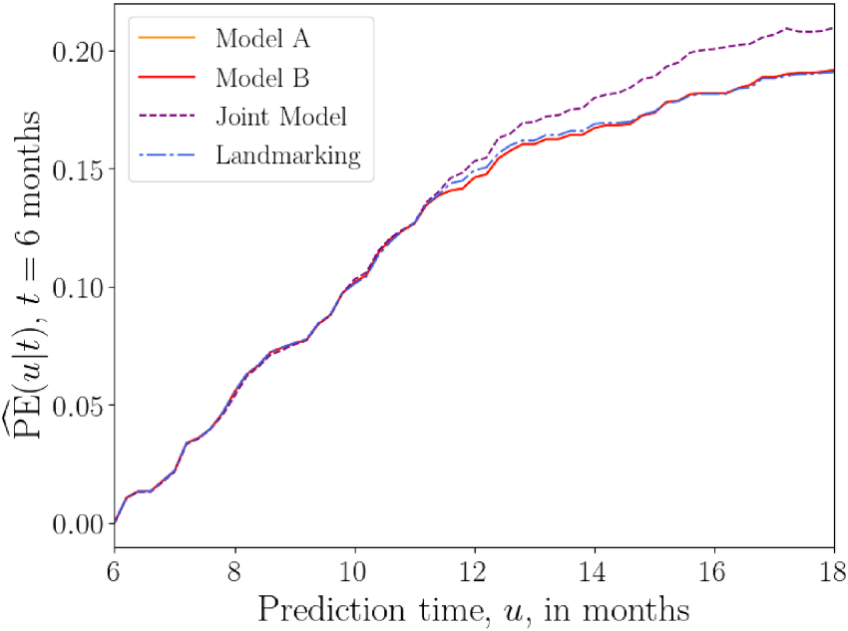
Overall prediction error $\hat{\mathrm{PE}}(u|t)$ plotted versus prediction time u (in months) for the AIDS data with fixed base time t=6 months. This error is calculated for u ranging from 6 to 18 months, at 0.2 month intervals. In equation (26), a squared loss function was used. The prediction error plotted at each time u is an average over values of $\hat{\mathrm{PE}}(u|t)$ calculated at each iteration of the 10-fold cross-validation procedure. The results from delayed kernel models A and B are plotted alongside the results from the landmarking model and a joint model. The results from model A cannot be seen because they overlap with the results from model B. PE: prediction error; AIDS: acquired immunodeficiency syndrome.

The plots of $\hat{\mathrm{PE}}(u|t)$ against prediction time u with base time t=6 months are shown in Figure 7 for the four models. As before, the data for $\hat{\mathrm{PE}}(u|t)$ is calculated from 10-fold cross-validation. All models show comparable accuracy up to u=11 months. After this time, the joint model shows slightly worse prediction than the other three (whose accuracies remain almost equal).

**Figure 8.**
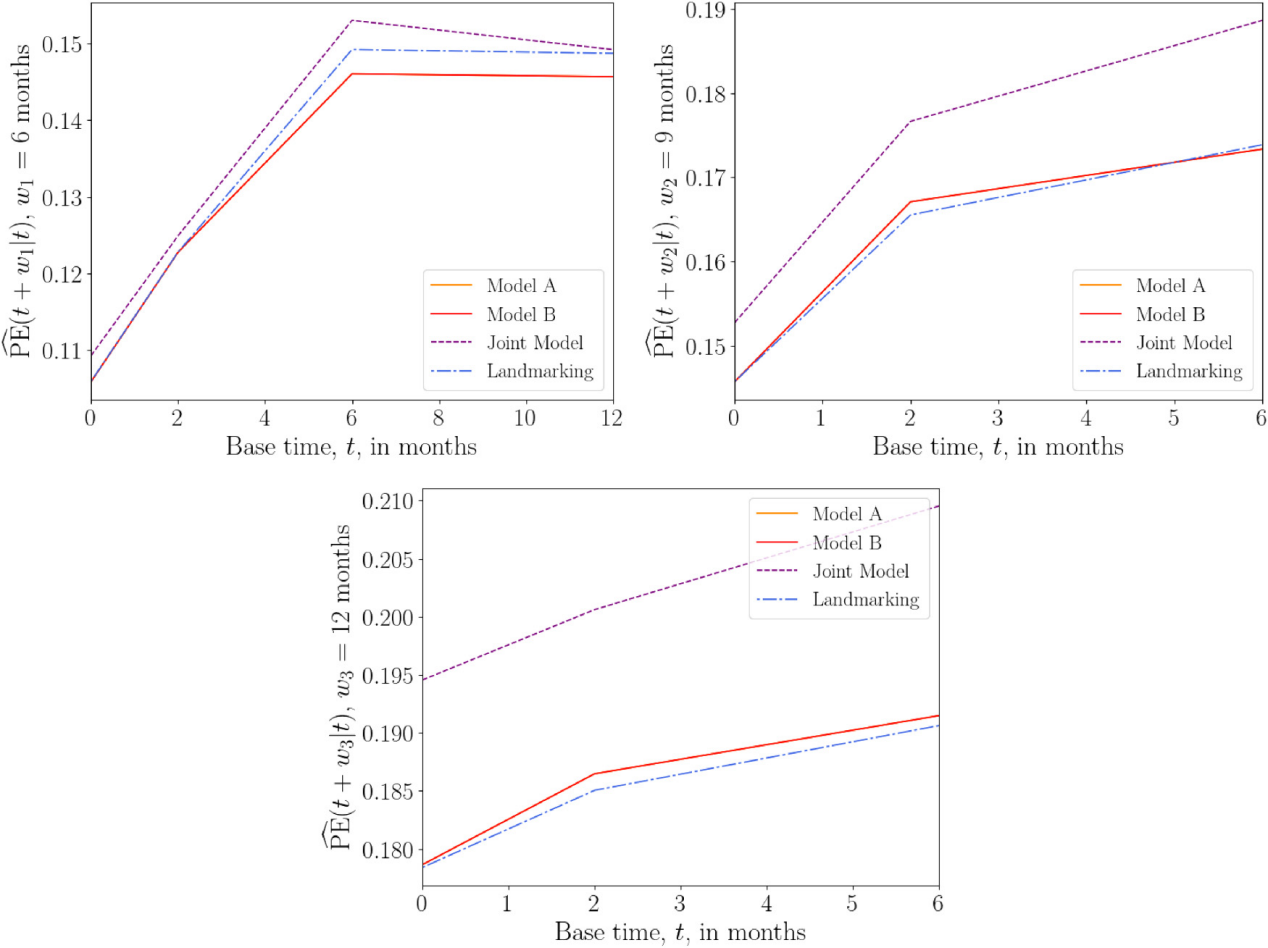
Overall prediction error $\hat{\mathrm{PE}}(u|t)$ versus base time t (in months) for the AIDS data with three fixed prediction windows: $w_{1}=6$ months, $w_{2}=9$ months and $w_{3}=12$ months. The prediction times are u=t+w . Observations are made at times 0, 2, 6, 12, and 18 months for all individuals in this data set. Prediction errors are hence only updated at these time points. For prediction window $w_{1}$ , prediction error is measured for t=0,2,6 and 12 months. For windows $w_{2}$ and $w_{3}$ , the error is measured at t=0,2 and 6 months only. In equation (26), we used a squared loss function. The prediction error plotted at each time t is an average over values of $\hat{\mathrm{PE}}(u|t)$ calculated at each iteration of the 10-fold cross-validation procedure. The results from delayed kernel models A and B are plotted alongside the landmarking model and a joint model. The results from model A cannot be seen clearly because they overlap with the results from model B. PE: prediction error; AIDS: acquired immunodeficiency syndrome.

Figure 8 shows plots of $\hat{\mathrm{PE}}(u|t)$ against base time for the AIDS data set with three prediction windows, $w_{1}=6$ months, $w_{2}=9$ months and $w_{3}=12$ months. For all prediction windows, the joint model has the highest prediction error. For the shortest prediction window $w_{1}$ , the DK and landmarking models have similar errors at t=0 and 2 months. After this, the DK models perform best. For the other prediction windows, these three models exhibit similar prediction errors.

The above results suggest that, for the AIDS data set, the joint model has the worst predictive accuracy overall, while the landmarking and DK models perform similarly.

### 4.4. Liver data set

For the liver data set, we model prothrombin index as our one longitudinal covariate $z_{1}^{i}(t)$ , and drug group as our single fixed covariate $\zeta_{1}^{i}$ . The fixed covariate is defined such that $\zeta_{1}^{i}=1$ for individuals in the treatment (prednisone) group, and $\zeta_{1}^{i}=0$ for those in the placebo group.

#### 4.4.1. Models

Following Rizopoulos,^16^ we define a flexible longitudinal model for the subject-specific prothrombin trajectories, using natural cubic splines with different average profiles for each drug group. Rizopoulos^16^ also suggests to include a separate indicator variable of the baseline measurement, to capture sudden changes in the prothrombin index in the early part of follow-up. The longitudinal model then takes the form

(37) $$\begin{array}{rl} z_{1}^{i}(t) & =m_{1}^{i}(t)+\varepsilon_{1}^{i}(t)\\ & =\eta_{1,0}+b_{1,0}^{i}+(\eta_{1,1}+b_{1,1}^{i})B_{1}^{n}(t,\lambda )+(\eta_{1,2}+b_{1,2}^{i})B_{2}^{n}(t,\lambda )+(\eta_{1,3}+b_{1,3}^{i})B_{3}^{n}(t,\lambda )\\ & \;+\eta_{1,4}\zeta_{1}^{i}B_{1}^{n}(t,\lambda )+\eta_{1,5}\zeta_{1}^{i}B_{2}^{n}(t,\lambda )+\eta_{1,6}\zeta_{1}^{i}B_{3}^{n}(t,\lambda )+\eta_{1,7}\zeta_{1}^{i}\\ & \;+\eta_{1,8}I(t=t_{i,1})+\eta_{1,9}\zeta_{1}^{i}I(t=t_{i,1})+\varepsilon_{1}^{i}(t) \end{array}$$

where $I(t=t_{i,1})$ is the indicator variable for the baseline time and, as before, $\left\{B_{k}^{n}(t,\lambda );k=1,2,3\right\}$ is the B-spline matrix for a natural cubic spline of time. This time, two internal knots are placed at 33% and 66.7% percentiles of the follow-up times. The random effects are assumed to have a diagonal covariance matrix.

The hazard functions for the joint model and the landmark model (with landmark time υ ) are then

(38) $$\begin{array}{rl} h^{\mathrm{JM}}(t|M_{\left[0,t\right]}^{i}) & =h_{0}(t)\exp\;\left\{\gamma_{1}\zeta_{1}^{i}+\alpha_{1}m_{1}^{i}(t)\right\} \end{array}$$

(39) $$\begin{array}{rl} h^{\mathrm{LM}}(t|Z^{i},\upsilon ) & =h_{0}(t|\upsilon )\exp\;\left\{\gamma_{1}\zeta_{1}^{i}+\alpha_{1}(\upsilon ){\tilde{z}}_{1}^{i}(\upsilon )\right\} \end{array}$$

For the DK models, we have

(40) $$h^{\mathrm{DK}}(t|Z_{\left[0,s_{i}\right]}^{i})=h_{0}(t)\exp\;\{\gamma_{1}\zeta_{1}^{i}+\int_{0}^{\min(s_{i},t)}\beta_{1}(t,t^{\prime },s_{i})z_{1}^{i}(t^{\prime })dt^{\prime }\}$$

#### 4.4.2. Results

**Figure 9.**
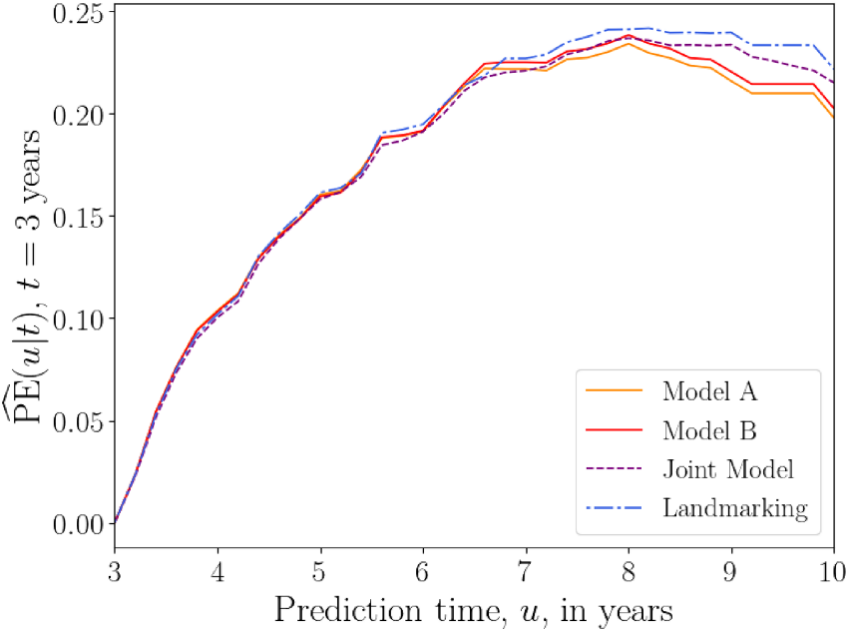
Overall prediction error $\hat{\mathrm{PE}}(u|t)$ plotted versus prediction time u (in years) for the Liver data with fixed base time t=3 years. This error is calculated for u ranging from 3 to 10 years, with 0.2 year increments. In equation (26) we used a squared loss function. The prediction error plotted at each time u is an average over values of $\hat{\mathrm{PE}}(u|t)$ calculated at each iteration of the 10-fold cross-validation procedure. The results from delayed kernel models A and B are plotted alongside the results from the landmarking model and a joint model.

Figure 9 shows the prediction error $\hat{\mathrm{PE}}(u|t)$ as a function of u for a fixed base time t=3 years for all four models. Again, each value of $\hat{\mathrm{PE}}(u|t)$ is obtained from the 10-fold cross-validation procedure. The four models show similar prediction error up to u=7 years. After this point, DK model A has the lowest prediction error, followed by DK model B, the joint model and finally, landmarking.

**Figure 10.**
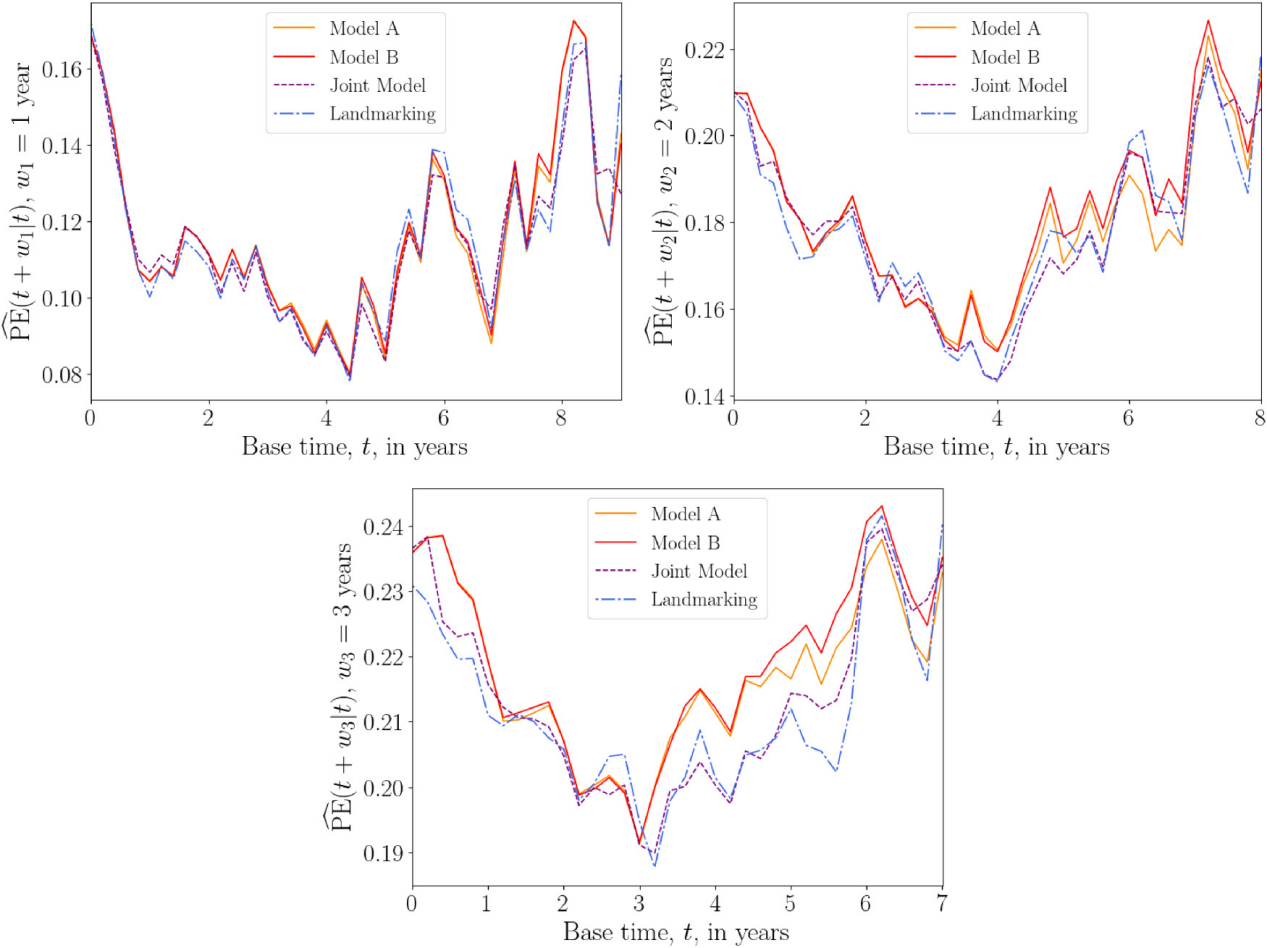
Overall prediction error $\hat{\mathrm{PE}}(u|t)$ plotted against base time t (in years) for the Liver data with three fixed prediction windows, $w_{1}=1$ year, $w_{2}=2$ years and $w_{3}=3$ years. The prediction times are u=t+w . The error is calculated for t ranging from 0 to 9,8 or 7 years, for $w_{1}$ , $w_{2}$ and $w_{3}$ respectively, with 0.2 year intervals. In equation (26) a squared loss function was used. The prediction error plotted at each time t is an average over values of $\hat{\mathrm{PE}}(u|t)$ calculated at each iteration of the 10-fold cross-validation procedure. The results from delayed kernel models A and B are plotted alongside the landmarking model and a joint model.

Plots of average $\hat{\mathrm{PE}}(u|t)$ against base time t are shown in Figure 10 for fixed prediction windows $w_{1}=1$ year, $w_{2}=2$ years and $w_{3}=3$ years. For all three windows, the four models exhibit very similar accuracy levels, with no model showing consistently superior predictions. For the largest prediction window $w_{3}$ , the DK models show slightly higher prediction errors compared with the standard methods between base times t=3 years and t=6 years.

For the liver data set, the above results suggest that the DK models have a predictive accuracy that is comparable to those of standard methods.

## 5. Summary and discussion

In this work, we propose a DK approach to dynamic prediction in survival analysis. In terms of complexity, our method comes somewhere between the two standard approaches, JM and landmarking. It is more parsimonious than JM, as it does not model the longitudinal covariate trajectory, and it makes no assumptions about the baseline hazard function. This makes the method more practical for data with multiple time-dependent covariates. The DK approach conditions only on the observed covariates and, unlike JM, makes no assumptions about covariate values in the future. This makes it more suitable for covariates that cannot easily be predicted, such as categorical ones. Compared to landmarking, the DK approach makes use of more of the available data. In landmarking, a new model is fitted at each landmark time, discarding individuals in the data set who have experienced the event before this landmark time. Additionally, standard landmarking only uses covariate measurements that are most recent before the landmark time. In contrast, the DK approach fits a single model that incorporates information from all individuals in the data set, using the full history of their covariate measurements. We note that in extensions of the landmarking approach one could, in principle, fit a landmarking model using multiple covariate measurements, for example, by replacing the most recent measurement with the mean value of measurements up to that time.

The DK approach relies on parameterization of the association kernels $\beta_{\mu }(t,t^{\prime },s)$ . In this work, we focused on two specific parameterizations, motivated by practical considerations. We required that our models reduce to the standard Cox model for static covariates, and that they contain the instantaneous Cox model as a special case, so that they are natural extensions of familiar models. However, alternative parameterizations or extensions (if demanded by the data at hand) can be incorporated without much effort. For example, one could include a “hard” time delay between covariate variations and their effect on hazard, or use parameterizations that favour time-translation invariance over consistency with standard Cox models. Furthermore, one need not be restricted to the exponential model proposed here but could instead make use of a more flexible parametric model for the decay.

In tests on medical data, we found that the DK approach performs similarly to the two standard approaches in terms of predictive accuracy. Depending on the data set, base time and prediction window, each method (JM, landmarking, or delayed kernels) had at some point the highest or the lowest prediction error; none appeared to be consistently superior or inferior across the scenarios we tested. An open question that should be the focus of future work is how to establish which longitudinal survival model will perform best for a given scenario. In simulations with data generated from joint models, the DK models had larger prediction errors than both joint models and landmarking. Here, landmarking even outperformed correctly specified joint models in some cases. Our results, therefore, suggest that the straightforward landmarking approach might still be a good choice in practice, while joint models suffer from mis-specification.

Although the DK models do not consistently outperform existing methods yet, we still see value in expressing a problem in a new formulation. We believe that our approach is less ‘ad-hoc’ than landmarking, and we expect that, in the future, suitable delayed kernels can be defined that reproduce the landmarking method. This would then significantly enrich the landmarking idea since many different kernels can be developed, and tailored to characteristics of the data sets at hand. In this spirit, we were careful to include standard models as special cases of our own.

There is scope to further develop the DK approach. For example, we used a naïve interpolation procedure (LOCF) but could try smoother interpolation methods such as Gaussian convolutions.^45,46^ We could also take a Bayesian inference approach, using non-informative prior distributions or incorporating existing knowledge into informative ones. While JM takes into account measurement errors, we have not attempted to do this for the DK model. Cox models were indeed found to be biased in the presence of such errors.^17,49,50^ Hence, future work could involve building measurement error effects into the DK approach. One could also build on methods from WCE models^37^ and use a wider class of association kernels, for example, those estimated via spline functions.

In this work, we compared against standard landmarking models, though extensions to these models exist.^2,33,51^ Similarly, in our analysis of real data sets we only considered joint models with instantaneous dependence on the ‘true’ covariate trajectory $m_{\mu }^{i}(t)$ in the hazard function. This study serves as a ‘proof of concept’ and as a starting point for future investigations; we leave systematic comparison of alternative model variants to future work. Such comparative research could benefit from recent developments in simulation methods for dynamic predictions with time-varying covariates.^11^ Generating data according to the DK model with dependence on the period over which covariates are observed is non-trivial, but could possibly be achieved by extending the permutational algorithm developed by Sylvestre and Abrahamowicz.^52^ Such simulations could provide valuable tests for internal consistency.

In summary, we have developed a ‘delayed kernel’ approach to dynamic prediction that overcomes some limitations of existing methods. By conditioning the hazard rate on observed covariates over a given time frame, it offers a simpler alternative to joint models without disregarding portions of longitudinal covariate data, as is the case with landmarking methods. Using three different clinical data sets we have demonstrated that DKs can have a predictive accuracy comparable to that of established methods. Therefore, we believe that the DK method is a promising addition to the toolbox of dynamic prediction methods.

## Supplemental Material

sj-pdf-1-smm-10.1177_09622802241275382 - Supplemental material for Delayed kernels for longitudinal survival analysis and dynamic prediction Supplemental material, sj-pdf-1-smm-10.1177_09622802241275382 for Delayed kernels for longitudinal survival analysis and dynamic prediction by Annabel Louisa Davies, Anthony CC Coolen and Tobias Galla in Statistical Methods in Medical Research

## Data Availability

Python and R codes used to perform the data analysis in this manuscript are available at the GitHub repository https://github.com/AnnieDavies/Supplement\_Davies\_Coolen\_Galla\_2024. The data sets analysed are available publicly via the JMbayes (and JMbayes2) R package.^25^
