# Supplementary material for: Delayed kernels for longitudinal survival analysis and dynamic prediction
Source: Stat Methods Med Res. 2024 Aug 30;33(10):1836–58. doi: 10.1177/09622802241275382 (PMC11577694; doi:10.1177/09622802241275382)
Supplement: sj-pdf-1-smm-10.1177_09622802241275382 - Supplemental material for Delayed kernels for longitudinal survival analysis and dynamic prediction [file sj-pdf-1-smm-10.1177_09622802241275382.pdf]

Delayed kernels for longitudinal survival analysis and dynamic prediction

—— Supplementary Material ——

Annabel L Davies[1,2], Anthony C C Coolen[3,4] and Tobias Galla[1,5]

S1 Visualization of delayed kernels

In this section we provide visualizations of the two delayed kernel models A and B. To do so we first restrict the parameter space appropriately and write our models in a simplified form.

By definition we always have $t' \leq \min(s,t)$. We focus here on the most relevant setting where $t' < s < t$. Following from Equations (16) and (17) in the main paper, the two kernels are defined as follows, in dimensionless form,

$$\beta^A(t,t',s)\frac{\tau}{a} = \frac{\mathrm{e}^{t'/\tau}}{\mathrm{e}^{s/\tau}-1}, \tag{S1}$$

$$\beta^B(t,t',s)\frac{\tau}{a} = \mathrm{e}^{-(t-t')/\tau} + \frac{\tau}{s}\Big\{1-\mathrm{e}^{-t/\tau}\Big(\mathrm{e}^{s/\tau}-1\Big)\Big\}. \tag{S2}$$

Upon defining $\tilde{s}=s/\tau$, $\tilde{t}'=t'/\tau$, and $\tilde{t}=t/\tau$, these expressions become

$$\tilde{\beta}^A(t,t',s) = \beta^A(t,t',s)\frac{\tau}{a} = \frac{\mathrm{e}^{\tilde{t}'}}{\mathrm{e}^{\tilde{s}}-1}, \tag{S3}$$

$$\tilde{\beta}^B(t,t',s) = \beta^B(t,t',s)\frac{\tau}{a} = \mathrm{e}^{\tilde{t}'-\tilde{t}} + \frac{1}{\tilde{s}}\Big[1-\mathrm{e}^{-\tilde{t}}\Big(\mathrm{e}^{\tilde{s}}-1\Big)\Big]. \tag{S4}$$

Let us evaluate some properties of these curves:

- Derivatives with respect to $\tilde{t}'$:

$$\frac{\partial\tilde{\beta}^A}{\partial\tilde{t}'} = \frac{\mathrm{e}^{\tilde{t}'}}{\mathrm{e}^{\tilde{s}}-1}, \qquad \frac{\partial\tilde{\beta}^B}{\partial\tilde{t}'} = \mathrm{e}^{\tilde{t}'-\tilde{t}} \tag{S5}$$

- Boundary values for t' are:

$$\beta^A(t,0,s)\frac{\tau}{a} = \frac{1}{\mathrm{e}^{\tilde{s}}-1}, \qquad \beta^A(t,s,s)\frac{\tau}{a} = \frac{1}{1-\mathrm{e}^{-\tilde{s}}} \tag{S6}$$

$$\beta^B(t,0,s)\frac{\tau}{a} = \mathrm{e}^{-\tilde{t}} + \frac{1}{\tilde{s}}\Big[1-\mathrm{e}^{-\tilde{t}}\Big(\mathrm{e}^{\tilde{s}}-1\Big)\Big], \qquad \beta^B(t,s,s)\frac{\tau}{a} = \mathrm{e}^{s-\tilde{t}} + \frac{1}{\tilde{s}}\Big[1-\mathrm{e}^{-\tilde{t}}\Big(\mathrm{e}^{\tilde{s}}-1\Big)\Big] \tag{S7}$$

- Boundary values for s are:

$$\beta^A(t,t',t')\frac{\tau}{a} = \frac{1}{1-\mathrm{e}^{-\tilde{t}'}}, \qquad \beta^A(t,t',t)\frac{\tau}{a} = \frac{\mathrm{e}^{\tilde{t}'}}{\mathrm{e}^{t}-1} \tag{S8}$$

$$\beta^B(t,t',t')\frac{\tau}{a} = \mathrm{e}^{\tilde{t}'-\tilde{t}} + \frac{1}{\tilde{t}'}\Big[1-\mathrm{e}^{-\tilde{t}}\Big(\mathrm{e}^{\tilde{t}'}-1\Big)\Big], \qquad \beta^B(t,t',t)\frac{\tau}{a} = \mathrm{e}^{\tilde{t}'-\tilde{t}} + \frac{1}{\tilde{t}}\mathrm{e}^{-\tilde{t}} \tag{S9}$$

[1]Department of Physics and Astronomy, University of Manchester, UK
[2]Department of Population Health Sciences, Bristol Medical School, University of Bristol, UK
[3]Department of Biophysics, Radboud University, The Netherlands
[4]Saddle Point Science Ltd, UK
[5]Instituto de Física Interdisciplinar y Sistemas Complejos, IFISC (CSIC-UIB), Campus Universitat Illes Balears, Palma de Mallorca, Spain

Corresponding author:
Annabel L Davies, Department of Population Health Sciences, Bristol Medical School, The University of Bristol, Bristol BS8 1QU, United Kingdom
Email: annabel.davies@bristol.ac.uk

Note that $\beta^A(t,t',t')\frac{\tau}{a}$ and $\beta^B(t,t',t')\frac{\tau}{a}$ are strictly non-negative.

Figure S1 shows model A (Eq (S1)) for three values of τ and various values of $s < t$ (where these kernels are independent of t). Here we observe, as expected, that covariates measured most recently before s have the largest contribution to the hazard. Figure S2 shows model B (Eq (S2)) for the same three values of τ, three values of t and various values of s (subject to $s < t$). Again, more recent measurements have an increasing influence on the hazard but now, for a covariate history that is exceedingly distant from the current time t ($s << t$ relative to τ) the kernel effectively describes a covariate time average (all measurements are given equal weighting).

The delayed kernel model for the Liver data is given in Equation (40) in the main text subject to the parameterization of the kernel $\beta(t,t',s)$ (i.e. model A or B). Fitting each model to the full Liver data set we obtain the following parameter estimates for model A

$$a = -0.027 \qquad \tau = 1.075 \qquad \gamma = -0.053, \tag{S10}$$

and for model B

$$a = -0.027 \qquad \tau = 0.885 \qquad \gamma = -0.056. \tag{S11}$$

The resulting kernels are plotted in Figure S3 assuming $s = 3$. For model B kernels are plotted for three values of t. Recall that γ is the association parameter for the fixed covariate representing the drug (treatment) group and therefore does not affect the form of the kernel $\beta(t,t',s)$.

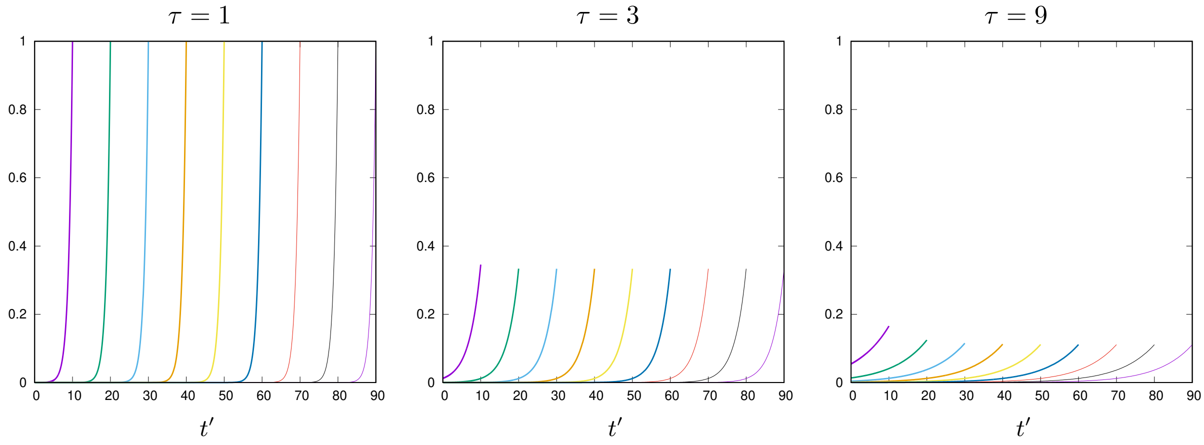

Figure S1. The kernels $\beta^A(t,t',s)/a$ plotted versus t', for $t > s$ (where these kernels are then independent of t, i.e. $\beta^A(t',s)/a$), and with $s \in \{10,20,30,40,50,60,70,80,90\}$ (from left to right in each figure). Note that by construction one always has $\beta^A(t',s) = 0$ for $t' > s$, and that $\int_0^s \mathrm{d}t'\ \beta^A(t',s) = 1$. This simple kernel integrates over all times up to s, decreasing exponentially with time separation. However, since it is independent of t it can represent the impact of past covariate events whose impact on instantaneous risk persists into the future.

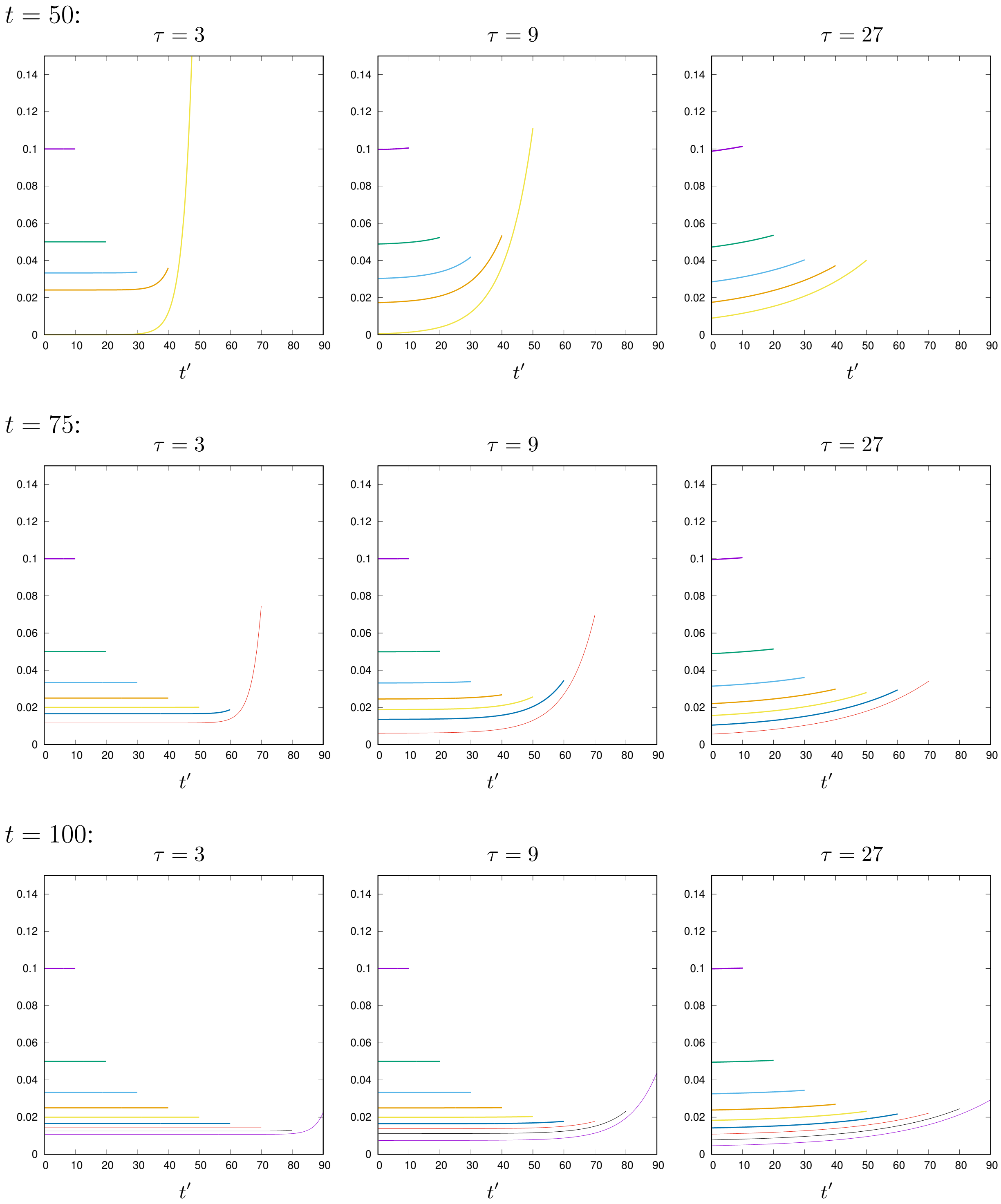

Figure S2. The kernels $\beta^B(t,t',s)/a$ plotted versus t', for the values $t \in \{50, 75, 100\}$ (top, middle, and bottom row), and with $s \in \{10, 20, 30, 40, 50, 60, 70, 80, 90\}$ (subject to $s < t$, from left to right in each figure). Note that by construction one always has $\beta^B(t,t',s) = 0$ for $t' > \min(s,t)$, and that $\int_0^{\min(s,t)} \mathrm{d}t'\ \beta^B(t,t',s) = 1$. Also this kernel integrates over all times up to s, decreasing with time separation, but now for the covariate history that is exceedingly distant from the current time t (relative to τ) the covariate time average is effectively used.

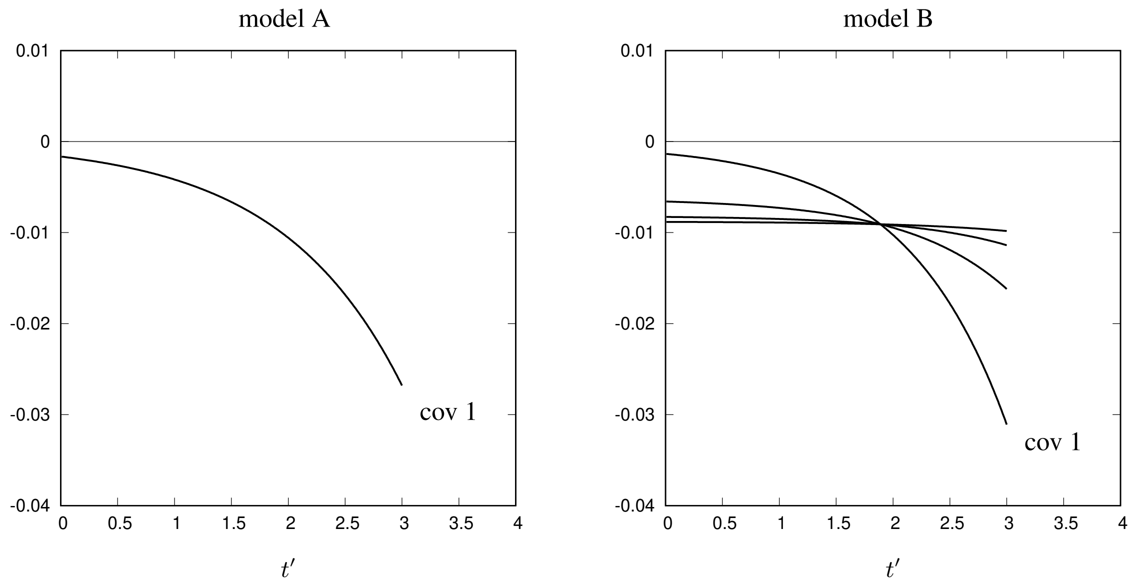

Figure S3. The kernels $\beta^A(t', s)$ (left figure) and $\beta^B(t, t', s)$ (right figure) plotted versus t' with the parameter values inferred for the Liver data set. In all cases we set $s = 3$ (in years), and there was just one longitudinal covariate. For model B curves are shown for $t \in \{3, 4, 5, 6\}$ (curves 'flatten out' for larger t). Both models infer that the most recent longitudinal values have a larger impact on the instantaneous hazard rates; in model B the time dependence gets stronger if the time t is closer to the final measurement time s.

S2 Mathematical details

S2.1 Maximum Likelihood Inference

As in standard Cox survival analysis, we use maximum likelihood inference to determine the most plausible values of the model parameters for our models, based on the observed data. We write θ for the full set of parameters, this includes the baseline hazard function $h_0(t)$. That is, $\theta = \{h_0(t), a_\mu, \tau_\mu; \mu = 1, \ldots, p\}$ for both models A and B. The optimal parameters are those for which the data likelihood $\mathcal{P}(\mathcal{D}|\theta)$ is maximized. We use the primary event indicator $\delta_i = I(T_i^* \leq C_i) \in \{0, 1\}$, where the indicator function $I(A)$ is defined as $I(A) = 1$ if A holds, and $I(A) = 0$ otherwise. The data likelihood for censored data is then

$$\mathcal{P}(\mathcal{D}|\theta) = \prod_{i=1}^{N} h(T_i|\theta, \mathcal{Z}^i_{[0,s_i]})^{\delta_i} S(T_i|\theta, \mathcal{Z}^i_{[0,s_i]}), \quad S(T_i|\theta, \mathcal{Z}^i_{[0,s_i]}) = \mathrm{e}^{-\int_0^{T_i} h(t|\theta, \mathcal{Z}^i_{[0,s_i]})\mathrm{d}t}, \tag{S12}$$

where, in this section, we refer only to the delayed kernel model and therefore omit the superscript 'DK' from the hazard for clarity. Maximizing $\mathcal{P}(\mathcal{D}|\theta)$ is equivalent to minimizing the negative log likelihood, i.e. $\hat{\theta}_{\mathrm{ML}} = \mathrm{argmin}_\theta\, \Omega_{\mathrm{ML}}(\theta)$, with

$$\begin{aligned} \Omega_{\mathrm{ML}}(\theta) &= -\log \prod_{i=1}^{N} h(T_i|\theta, \mathcal{Z}^i_{[0,s_i]})^{\delta_i} S(T_i|\theta, \mathcal{Z}^i_{[0,s_i]}) \\ &= -\sum_{i=1}^{N} \delta_i \log h(T_i|\theta, \mathcal{Z}^i_{[0,s_i]}) + \sum_{i=1}^{N} \int_0^{T_i} h(t|\theta, \mathcal{Z}^i_{[0,s_i]})\mathrm{d}t. \end{aligned} \tag{S13}$$

The hazard rate for the delayed kernel approach is

$$h(t|\theta, \mathcal{Z}^i_{[0,s_i]}) = h_0(t) \exp\left\{ \sum_{\mu=1}^{p} \int_0^{\min(s_i,t)} \beta_\mu(t, t', s_i) z^i_\mu(t')\mathrm{d}t' \right\}. \tag{S14}$$

Substituting this into $\Omega_{\rm ML}(\theta)$ yields

$$\Omega_{\rm ML}(\theta) = -\sum_{i=1}^{N}\delta_i \log h_0(T_i) - \sum_{i=1}^{N}\delta_i \int_0^{s_i}\sum_{\mu}\beta_\mu(T_i,t',s_i)z_\mu^i(t'){\rm d}t' + \sum_{i=1}^{N}\int_0^{T_i} h_0(t'){\rm e}^{\int_0^{\min(s_i,t')}{\rm d}t''\sum_\mu \beta_\mu(t',t'',s_i)z_\mu^i(t'')}{\rm d}t' \,, \tag{S15}$$

where we have used the fact that $s_i \le T_i$. For simplicity we have specified the hazard in Equation (S15) without fixed (or baseline) covariates. To include these explicitly (if desired), one can simply add the term $\sum_\nu \gamma_\nu \zeta_\nu^i$ to the exponent of the hazard function.

Extremization of Equation (S15) functionally over $h_0(t)$ gives the maximum likelihood estimator of the baseline hazard function, given $\beta_\mu(t,t',s)$,

$$\hat{h}_0(t) = \frac{\sum_{i=1}^{N}\delta_i\delta(t-T_i)}{\sum_{i=1}^{N} I(t\in[0,T_i]){\rm e}^{\int_0^{\min(s_i,t)}\sum_\mu\beta_\mu(t,t',s_i)z_\mu^i(t'){\rm d}t'}}, \tag{S16}$$

as quoted in Equation (21) in the main paper. Equation (S16) is the analogue of the standard Breslow estimator.[1] Inserting this expression back into Equation (S15) leaves the following function to be extremized over the remaining model parameters $\{a_\mu,\tau_\mu\}$ in the kernels $\beta_\mu(t,t',s)$ (where we denote all terms that do not contain $\{a_\mu,\tau_\mu\}$ simply as 'constant'):

$$\begin{aligned}
\Omega_{\rm ML}[\{a_\mu,\tau_\mu\}] &= -\sum_{i=1}^{N}\delta_i \log\left(\frac{\sum_{k=1}^{N}\delta_k\delta(T_i-T_k)}{\sum_{j=1}^{N} I(T_i\in[0,T_j]){\rm e}^{\sum_\mu\int_0^{\min(s_j,T_i)}\beta_\mu(T_i,t',s_j)z_\mu^j(t'){\rm d}t'}}\right) \\
&\quad -\sum_{i=1}^{N}\delta_i\sum_\mu\int_0^{s_i}\beta_\mu(T_i,t',s_i)z_\mu^i(t'){\rm d}t' \\
&\quad +\sum_{i=1}^{N}\int_0^{T_i}\left(\frac{\sum_{k=1}^{N}\delta_k\delta(t'-T_k)}{\sum_{j=1}^{N} I(t'\in[0,T_j]){\rm e}^{\sum_\mu\int_0^{\min(s_j,t')}\beta_\mu(t',t'',s_j)z_\mu^j(t''){\rm d}t''}}\right){\rm e}^{\sum_\mu\int_0^{\min(s_i,t')}\beta_\mu(t',t'',s_i)z_\mu^i(t''){\rm d}t''}{\rm d}t' \\
&= \sum_{i=1}^{N}\delta_i\left\{\log\left(\sum_{j=1}^{N} I(T_i\in[0,T_j]){\rm e}^{\sum_\mu\int_0^{\min(s_j,T_i)}\beta_\mu(T_i,t',s_j)z_\mu^j(t'){\rm d}t'}\right) - \sum_\mu\int_0^{s_i}\beta_\mu(T_i,t',s_i)z_\mu^i(t'){\rm d}t'\right\} \\
&\quad +\sum_{k=1}^{N}\delta_k\left(\frac{\sum_{i=1}^{N} I(T_k\in[0,T_i]){\rm e}^{\sum_\mu\int_0^{\min(s_i,T_k)}\beta_\mu(T_k,t'',s_i)z_\mu^i(t''){\rm d}t''}}{\sum_{j=1}^{N} I(T_k\in[0,T_j]){\rm e}^{\sum_\mu\int_0^{\min(s_j,T_k)}\beta_\mu(T_k,t'',s_j)z_\mu^j(t''){\rm d}t''}}\right) + \text{constant} \\
&= \sum_{i=1}^{N}\delta_i\left\{\log\left(\sum_{j=1}^{N} I(T_i\in[0,T_j]){\rm e}^{\sum_\mu\int_0^{\min(s_j,T_i)}\beta_\mu(T_i,t',s_j)z_\mu^j(t'){\rm d}t'}\right) - \sum_\mu\int_0^{s_i}\beta_\mu(T_i,t',s_i)z_\mu^i(t'){\rm d}t'\right\} \\
&\quad + \text{constant}.
\end{aligned} \tag{S17}$$

This is the formula quoted in Equation (22) in the main paper. Minimization of Equation (S17) with respect to the remaining model parameters $\{a_\mu,\tau_\mu;\mu=1\ldots p\}$ must be performed numerically. Finally, if we define the N^2 integrals

$$\mathcal{I}_{ij}[\{a_\mu,\tau_\mu\}] = \int_0^{\min(s_j,T_i)}\sum_{\mu=1}^{p}\beta_\mu(T_i,t',s_j)z_\mu^j(t'){\rm d}t', \tag{S18}$$

then we can re-write expression (S17) as

$$\Omega_{\rm ML}[\{a_\mu,\tau_\mu\}] = \sum_{i=1}^{N}\delta_i\log\left(\sum_{j=1}^{N} I(T_i\in[0,T_j])\,{\rm e}^{\mathcal{I}_{ij}[\{a_\mu,\tau_\mu\}]-\mathcal{I}_{ii}[\{a_\mu,\tau_\mu\}]}\right) + \text{constant}. \tag{S19}$$

S2.2 Survival probability

We recall from Equation (23) in the main paper that the estimated probability that subject i has not experienced an event by time $u > s_i$ conditional on their survival to s_i and on their covariate values $\mathcal{Z}^i$ up to that time is given

by

$$\hat{\pi}^{\rm DK}(u|\mathcal{Z}^i_{[0,s_i]},s_i) = \exp\left\{-\int_{s_i}^{u}\hat{h}(t'|\mathcal{Z}^i_{[0,s_i]}){\rm d}t'\right\}. \tag{S20}$$

Substituting into this equation the baseline hazard estimator in Equation (S16), in combination with Equation (S14), yields

$$\begin{aligned}
\hat{\pi}^{\rm DK}(u|\mathcal{Z}^i_{[0,s_i]},s_i) &= \exp\left\{-\int_{s_i}^{u}\frac{{\rm e}^{\sum_{\mu=1}^{p}\int_0^{\min(s_i,t')}\hat{\beta}_\mu(t',t'',s_i)z^i_\mu(t''){\rm d}t''}\sum_{j=1}^{N}\delta_j\delta(t'-T_j)}{\sum_{k=1}^{N}I(t'\in[0,T_k]){\rm e}^{\int_0^{\min(s_k,t')}\sum_\mu\hat{\beta}_\mu(t',t'',s_k)z^k_\mu(t''){\rm d}t''}}{\rm d}t'\right\}\\
&= \exp\left\{-\sum_{j=1}^{N}\delta_j\, I(T_j\in[s_i,u])\frac{{\rm e}^{\sum_{\mu=1}^{p}\int_0^{\min(s_i,T_j)}\hat{\beta}_\mu(T_j,t'',s_i)z^i_\mu(t''){\rm d}t''}}{\sum_{k=1}^{N}I(T_j\in[0,T_k]){\rm e}^{\int_0^{\min(s_k,T_j)}\sum_\mu\hat{\beta}_\mu(T_j,t'',s_k)z^k_\mu(t''){\rm d}t''}}\right\}\\
&= \exp\left\{-\sum_{j=1}^{N}\delta_j\, I(T_j\in[s_i,u])\frac{{\rm e}^{\sum_{\mu=1}^{p}\int_0^{s_i}\hat{\beta}_\mu(T_j,t'',s_i)z^i_\mu(t''){\rm d}t''}}{\sum_{k=1}^{N}I(T_j\in[0,T_k]){\rm e}^{\int_0^{\min(s_k,T_j)}\sum_\mu\hat{\beta}_\mu(T_j,t'',s_k)z^k_\mu(t''){\rm d}t''}}\right\},
\end{aligned} \tag{S21}$$

where in the last line we replaced $\min(s_i,T_j)=s_i$, which holds by virtue of the factor $I(T_j\in[s_i,u])$. We have also used the notation $\hat{\beta}_\mu(t,t',s)$ to indicate the association kernel obtained from the ML estimators of the parameters $\{a_\mu,\tau_\mu\}$. Using the integral defined in Equation (S18) we can re-write Equation (S21) as

$$\hat{\pi}^{\rm DK}(u|\mathcal{Z}^i_{[0,s_i]},s_i) = \exp\left\{-\sum_{j=1}^{N}\delta_j\, I(T_j\in[s_i,u])\frac{{\rm e}^{\mathcal{I}_{ji}[\{\hat{a}_\mu\hat{\tau}_\mu\}]}}{\sum_{k=1}^{N}I(T_j\in[0,T_k]){\rm e}^{\mathcal{I}_{jk}[\{\hat{a}_\mu\hat{\tau}_\mu\}]}}\right\}, \tag{S22}$$

where we recall that i labels the individual for whom we are making predictions, while the sums over j and k refer to individuals in the data set used for inference.

S2.3 Last observation carried forward

In the main paper we use last observation carried forward (LOCF) as a straightforward method to interpolate between discrete measurements of the covariates. Therefore we set $z^i_\mu(t)=z^i_\mu(t_{i\ell})$ where $t_{i\ell}$ is the latest observation time before (or equal to) t, that is $\max\{t_{i\ell}:t_{i\ell}\le t\}$. The approximated continuous time covariate trajectory is then,

$$z^i_\mu(t)=\sum_{\ell=1}^{n_i-1}I(t\in[t_{i\ell},t_{i\ell+1}])z^i_\mu(t_{i\ell}). \tag{S23}$$

Using Equation (S23) along with the parameterizations of the association kernels, we can evaluate the integral in Equation (S18) analytically. We do this in the following sections for delayed kernel models A and B.

S2.3.1 Model A. We recall from Equation (16) in the main paper that the association kernel for model A is defined as

$$\beta_\mu(t,t',s)=\frac{a_\mu}{\tau_\mu}\frac{\exp(t'/\tau_\mu)}{\exp(\min(s,t)/\tau_\mu)-1}. \tag{S24}$$

Therefore, using the step function defined by $\theta(z>0)=1$ and $\theta(z<0)=0$, we have

$$\begin{aligned}
\mathcal{I}^{(A)}_{ij}[\{a_\mu,\tau_\mu\}] &= \sum_{\mu=1}^{p}\sum_{\ell=1}^{n_j-1}\frac{a_\mu z^j_\mu(t_{j\ell})}{{\rm e}^{\min(s_j,T_i)/\tau_\mu}-1}\int_0^{\min(s_j,T_i)}\frac{1}{\tau_\mu}{\rm e}^{t'/\tau_\mu}I(t'\in[t_{j\ell},t_{j\ell+1}]){\rm d}t'\\
&= \sum_{\mu=1}^{p}\sum_{\ell=1}^{n_j-1}\frac{a_\mu z^j_\mu(t_{j\ell})}{{\rm e}^{\min(s_j,T_i)/\tau_\mu}-1}\theta(\min(s_j,T_i,t_{j\ell+1})-t_{j\ell})\left[{\rm e}^{t'/\tau_\mu}\right]_{t_{j\ell}}^{\min(s_j,T_i,t_{j\ell+1})}\\
&= \sum_{\mu=1}^{p}\sum_{\ell=1}^{n_j-1}a_\mu z^j_\mu(t_{j\ell})\,\theta(\min(s_j,T_i,t_{j\ell+1})-t_{j\ell})\frac{{\rm e}^{\min(s_j,T_i,t_{j\ell+1})/\tau_\mu}-{\rm e}^{t_{j\ell}/\tau_\mu}}{{\rm e}^{\min(s_j,T_i)/\tau_\mu}-1}\\
&= \sum_{\mu=1}^{p}\sum_{\ell=1}^{n_j-1}a_\mu z^j_\mu(t_{j\ell})\,\theta(T_i-t_{j\ell})\frac{{\rm e}^{\min(T_i,t_{j\ell+1})/\tau_\mu}-{\rm e}^{t_{j\ell}/\tau_\mu}}{{\rm e}^{\min(s_j,T_i)/\tau_\mu}-1}
\end{aligned} \tag{S25}$$

where in the last line we used the fact that $t_{j\ell}<t_{j\ell+1}\le s_j$.

S2.3.2 Model B. We recall from Equation (17) in the main paper that the association kernel for model B is defined as

$$\beta_\mu(t,t',s) \;=\; \frac{a_\mu}{\tau_\mu}\mathrm{e}^{-(t-t')/\tau_\mu} + \frac{a_\mu}{\min(s,t)}\left[1-\mathrm{e}^{[\min(s,t)-t]/\tau_\mu}+\mathrm{e}^{-t/\tau_\mu}\right]. \tag{S26}$$

Substituting this into Equation (S18) gives

$$\begin{aligned}
\mathcal{I}^{(\mathrm{B})}_{ij}[\{a_\mu,\tau_\mu\}] \;&=\; \sum_{\mu=1}^{p}\sum_{\ell=1}^{n_j-1} a_\mu z^j_\mu(t_{j\ell})\mathrm{e}^{-T_i/\tau_\mu}\int_0^{\min(s_j,T_i)} I(t'\in[t_{j\ell},t_{j\ell+1}])\frac{1}{\tau_\mu}\mathrm{e}^{t'/\tau_\mu}\mathrm{d}t' \\
&+\sum_{\mu=1}^{p}\sum_{\ell=1}^{n_j-1} a_\mu z^j_\mu(t_{j\ell})\left(\int_0^{\min(s_j,T_i)} I(t'\in[t_{j\ell},t_{j\ell+1}])\mathrm{d}t'\right)\left(\frac{\mathrm{e}^{-T_i/\tau_\mu}}{\min(s_j,T_i)}+\theta(T_i-s_j)\frac{1-\mathrm{e}^{(s_j-T_i)/\tau_\mu}}{s_j}\right) \\
&= \sum_{\mu=1}^{p}\sum_{\ell=1}^{n_j-1} a_\mu z^j_\mu(t_{j\ell})\theta(\min(s_j,T_i,t_{j\ell+1})-t_{j\ell})\Bigg\{\mathrm{e}^{-T_i/\tau_\mu}\left(\mathrm{e}^{\min(s_j,T_i,t_{j\ell+1})/\tau_\mu}-\mathrm{e}^{t_{j\ell}/\tau_\mu}\right) \\
&\qquad +\Big(\min(s_j,T_i,t_{j\ell+1})-t_{j\ell}\Big)\left(\frac{\mathrm{e}^{-T_i/\tau_\mu}}{\min(s_j,T_i)}+\theta(T_i-s_j)\frac{1-\mathrm{e}^{(s_j-T_i)/\tau_\mu}}{s_j}\right)\Bigg\} \\
&= \sum_{\mu=1}^{p}\sum_{\ell=1}^{n_j-1} a_\mu z^j_\mu(t_{j\ell})\theta(T_i-t_{j\ell})\Bigg\{\mathrm{e}^{-T_i/\tau_\mu}\left(\mathrm{e}^{\min(T_i,t_{j\ell+1})/\tau_\mu}-\mathrm{e}^{t_{j\ell}/\tau_\mu}\right) \\
&\qquad +\Big(\min(T_i,t_{j\ell+1})-t_{j\ell}\Big)\left(\frac{\mathrm{e}^{-T_i/\tau_\mu}}{\min(s_j,T_i)}+\theta(T_i-s_j)\frac{1-\mathrm{e}^{(s_j-T_i)/\tau_\mu}}{s_j}\right)\Bigg\},
\end{aligned} \tag{S27}$$

where in the last line, we have again used the property $t_{j\ell} < t_{j\ell+1} \le s_j$.

S2.4 Estimating the hazard function

We obtain an estimate for the hazard function by substituting the maximum likelihood estimates $\{\hat{a}_\mu,\hat{\tau}_\mu\}$ into Equations (S16) and (S14). To evaluate these expressions we must perform the following integral

$$\mathcal{J}_i[t,\{a_\mu,\tau_\mu\}] = \int_0^{\min(s_i,t)}\sum_{\mu=1}^{p}\beta_\mu(t,t',s_i)z^i_\mu(t')\mathrm{d}t'. \tag{S28}$$

This is the same as integral $\mathcal{I}_{ij}[\{a_\mu,\tau_\mu\}]$ but replacing j with i and T_i with t. The maximum likelihood estimate of the hazard function can then be written as

$$\hat{h}(t|\hat{\theta},\mathcal{Z}^i_{[0,s_i]}) = \frac{\sum_{j=1}^N \delta_j\delta(t-T_j)}{\sum_{j=1}^N I(t\in[0,T_j])\mathrm{e}^{\mathcal{J}_j[t,\{\hat{a}_\mu,\hat{\tau}_\mu\}]}}\mathrm{e}^{\mathcal{J}_i[t,\{\hat{a}_\mu,\hat{\tau}_\mu\}]}, \tag{S29}$$

where, following the same procedures as in Sections S2.3.1 and S2.3.2, we find for Model A,

$$\mathcal{J}^{(\mathrm{A})}_i[t,\{a_\mu,\tau_\mu\}] = \sum_{\mu=1}^{p}\sum_{\ell=1}^{n_i-1} a_\mu z^i_\mu(t_{i\ell})\,\theta(t-t_{i\ell})\frac{\mathrm{e}^{\min(t,t_{i\ell+1})/\tau_\mu}-\mathrm{e}^{t_{i\ell}/\tau_\mu}}{\mathrm{e}^{\min(s_i,t)/\tau_\mu}-1}, \tag{S30}$$

and for Model B,

$$\begin{aligned}
\mathcal{J}^{(\mathrm{B})}_i[t,\{a_\mu,\tau_\mu\}] &= \sum_{\mu=1}^{p}\sum_{\ell=1}^{n_i-1} a_\mu z^i_\mu(t_{i\ell})\theta(t-t_{i\ell})\Bigg\{\mathrm{e}^{-t/\tau_\mu}\left(\mathrm{e}^{\min(t,t_{i\ell+1})/\tau_\mu}-\mathrm{e}^{t_{i\ell}/\tau_\mu}\right) \\
&\qquad + \Big(\min(t,t_{i\ell+1})-t_{i\ell}\Big)\left(\frac{\mathrm{e}^{-t/\tau_\mu}}{\min(s_i,t)}+\theta(t-s_i)\frac{1-\mathrm{e}^{(s_i-t)/\tau_\mu}}{s_j}\right)\Bigg\}.
\end{aligned} \tag{S31}$$

S3 Simulation study

Here we give details of the methods and results of the simulation study referenced in Section 3.5 of the main text. Synthetic data was generated from joint models in two scenarios: (1) with an instantaneous association function in the survival sub-model and (2) with a cumulative association.

S3.1 Methods

S3.1.1 Data generation model. For both scenarios 1 and 2 we generate longitudinal measurements from a simple linear random slopes and random intercepts model,

$$y_i(t) = \beta_0 + \beta_1 t + b_{i0} + b_{i1} t + \epsilon_i(t), \tag{S32}$$

where $\epsilon_i(t) \sim \mathcal{N}(0, \sigma^2)$ and $\boldsymbol{b} \sim \mathcal{N}(0, \boldsymbol{D})$. In order to simulate a realistic data set, we choose parameter values loosely based on the real data set and simulations performed in Rizopolous (2017).[2] For the longitudinal sub-model we use

$$\beta_0 = 2, \quad \beta_1 = 1.25 \tag{S33}$$

$$\sigma = 0.5, \boldsymbol{D} = \begin{pmatrix} 0.55 & 0.175 \\ 0.175 & 0.45 \end{pmatrix}. \tag{S34}$$

In both scenarios we sample event times according to a survival sub-model with a Weibull baseline hazard function,

$$h_0(t) = \exp(\gamma_0)\sigma_t t^{\sigma_t - 1}, \tag{S35}$$

and one binary variable x (representing the treatment group). In scenario 1, the hazard function depends on the instantaneous value of the longitudinal covariate,

$$h_i^{(1)}(t) = h_0(t) \exp\left\{\gamma_1 x + \alpha m_i(t)\right\}, \tag{S36}$$

and we choose the true parameter values

$$\alpha = 0.5, \quad \gamma_0 = -5.0, \quad \gamma_1 = 0.5, \quad \sigma_t = 0.95. \tag{S37}$$

The survival sub-model in scenario 2 is similar but instead has a cumulative association function,

$$h_i^{(2)}(t) = h_0(t) \exp\left\{\gamma_1 x + \int_0^t \alpha m_i(t')\mathrm{d}t'\right\}, \tag{S38}$$

and true parameters,

$$\alpha = 0.25, \quad \gamma_0 = -7.0, \quad \gamma_1 = 0.25, \quad \sigma_t = 0.95. \tag{S39}$$

S3.1.2 Data generation procedure. The code used to perform the simulation was modified from code published in Rizopolous (2017).[2] At each iteration of the simulation, data was generated for 1000 subjects who are followed up for a period of 15 years and have measurements planned at baseline and at 14 follow up times.

For each subject we set the baseline time point equal to zero and sample 14 follow up times uniformly between 0 and 15 years. We assign subjects to one of two treatment groups $x \in \{0, 1\}$ with equal probability and sample the random effects from $\boldsymbol{b} \sim \mathcal{N}(0, \boldsymbol{D})$. We use the sampled follow up times and random effects to generate 14 longitudinal measurements from Equation (S32).

To generate event times for each subject i, we sample a probability u_i from a uniform distribution $\mathrm{Unif}(0, 1)$ and set it equal to the survival probability

$$S(t) = \exp\left\{-\int_0^t h(t')\mathrm{d}t'\right\}, \tag{S40}$$

where $h(t)$ is the hazard function for the relevant scenario (either Equation (S36) or (S38)). Rearranging this equation gives

$$\ln u_i + \int_0^t h(t')\mathrm{d}t' = 0. \tag{S41}$$

We solve this equation for t using the R function *uniroot* and set this equal to the event time T_i^* (if a root cannot be found then this subject is removed from the data set). Next, we sample a censoring time C_i from a uniform distribution (with mean 14 for $x_i = 1$ and mean 10 for $x_i = 0$). The observed event time is then set equal to $T_i = \min(T_i^*, C_i)$.

Finally, all follow up times and longitudinal observations after time T_i are removed from the data set.

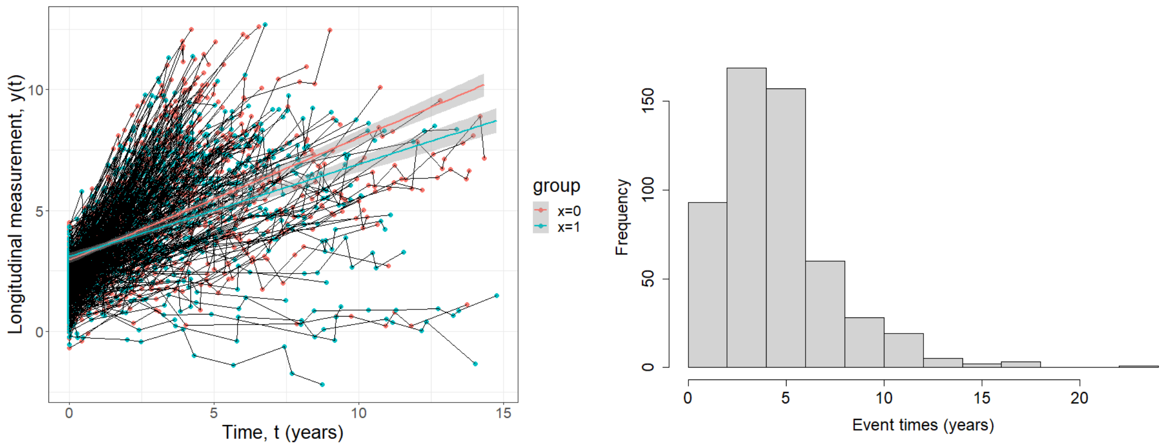

(a) Longitudinal trajectories. **(b)** Event times.

Figure S4. An example of simulated data for scenario 1 (instantaneous association).

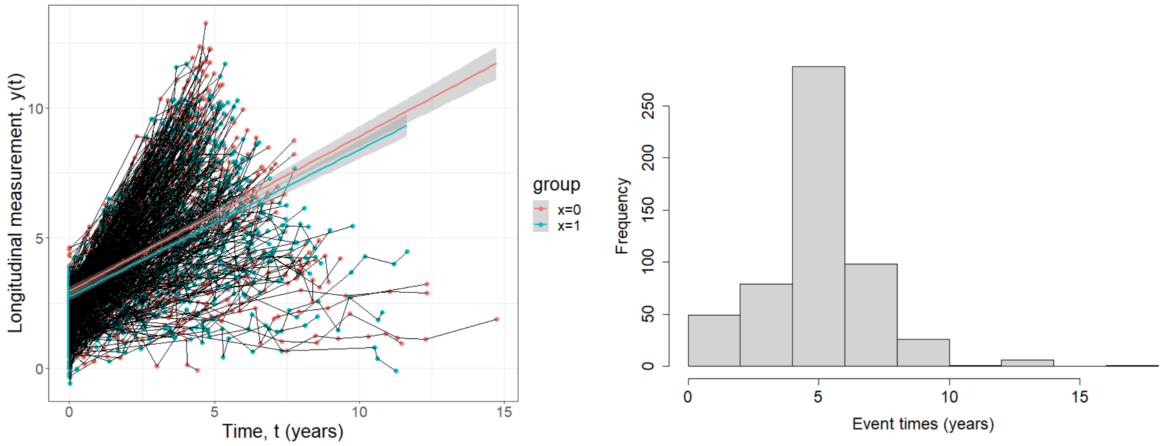

(a) Longitudinal trajectories. **(b)** Event times.

Figure S5. An example of simulated data for scenario 2 (cumulative association).

S3.1.3 Evaluating models. At each iteration, we evaluated the models using the same 10-fold cross-validation procedure described in Section 4.1 of the main paper. Figure S4 shows an example of the longitudinal trajectories and histogram of event times generated under scenario 1. Figure S5 shows the equivalent plots for scenario 2. Based on the simulated event times and the simulation study in Rizopolous (2017),[2] we use a prediction window of $w = 2$ years and five base times $t = (1.5, 3.5, 5.5, 7.5, 9.5)$.

For each scenario we fit two joint models, a landmarking model, and the delayed kernel models A and B. Both joint models have a correctly specified longitudinal sub-model (Equation (S32)). One joint model has an instantaneous association in the survival sub model (Equation (S36)) and the other has a cumulative association (Equation (S38)). Therefore each scenario is tested with a correctly specified joint model and a (partly) misspecified joint model. The joint models are fitted using the JMbayes R package.

S3.2 Results

We repeated the simulation 50 times. At each iteration we obtained (i) parameter estimates from fitting the delayed kernel and joint models to the full dataset, and (ii) prediction errors from cross validation associated with each base time (for all five models). The computational burden of implementing both the delayed kernel and joint models prevented us from increasing the number of iterations. However, Figure S6 shows that, except for at the first base time in scenario 1, the standard deviations in prediction errors across iterations were approximately stable after 50 iterations.

S3.2.1 Parameter estimates. Below we list the mean and SD of parameters estimated by the two joint models for scenario 1. Joint model 1 (JM1) has an instantaneous association and is correctly specified. Joint model 2 (JM2) has a cumulative association and is incorrectly specified. The true values of the parameters used to generate

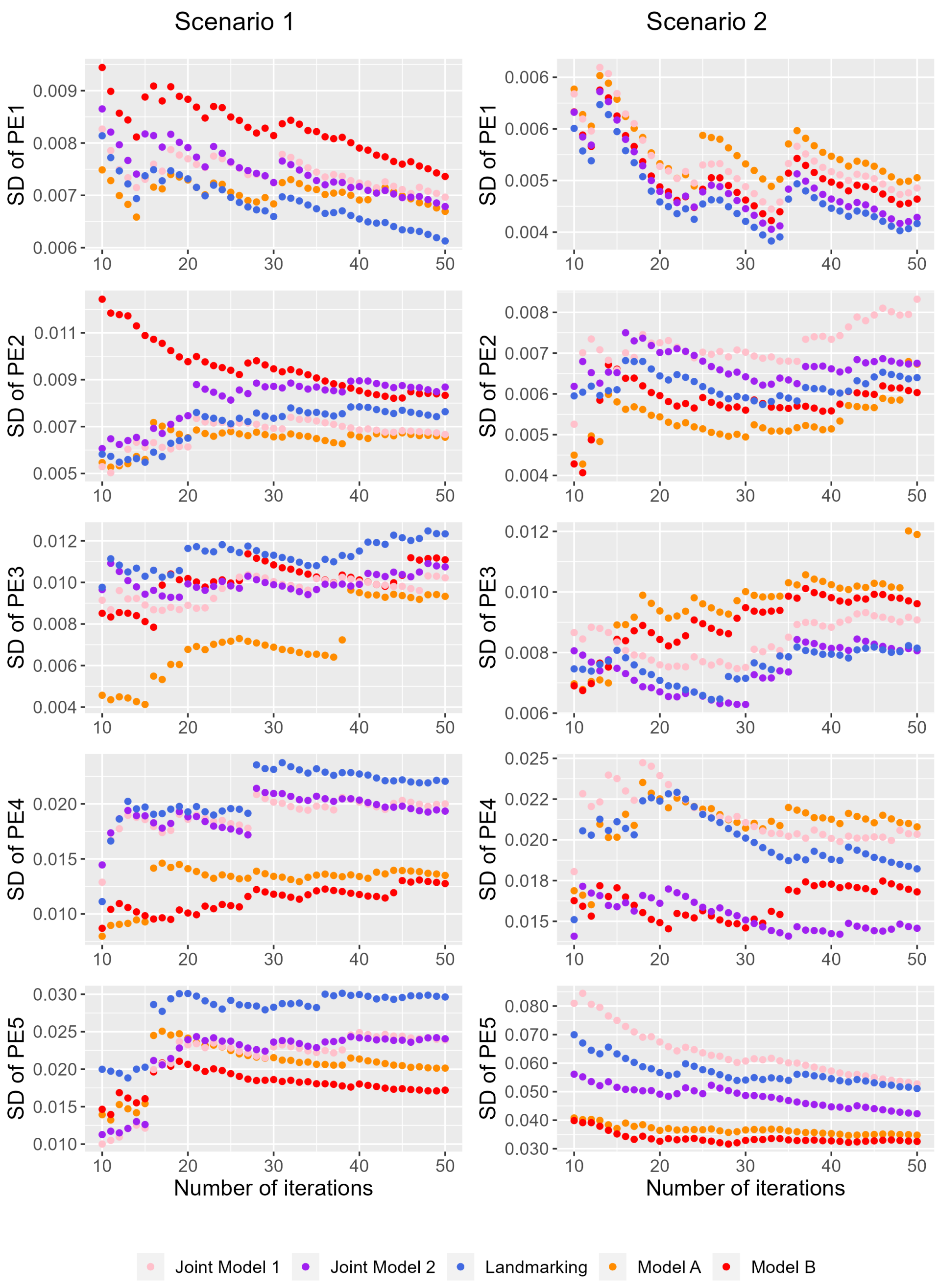

Figure S6. Plots showing the change in standard deviation of the prediction errors over the 50 iterations of the simulation. Results for simulated scenario 1 are shown on the left, and scenario 2 is on the right. Each row of plots corresponds to a different base time; i.e., PE1 is the prediction error calculated using a base time 1.5, PE2 is the prediction error for $t = 3.5$, PE3 is for $t = 5.5$, PE4 is for $t = 7.5$ and PE5 is for $t = 9.5$.

the data from joint model 1 are $\alpha = 0.5$ and $\gamma = 0.5$.

	$\alpha = 0.5$		$\gamma_1 = 0.5$	
	Mean	(SD)	Mean	(SD)
JM1 (correct):	0.492	(0.022)	0.486	(0.089)
JM2 (incorrect):	0.142	(0.010)	0.472	(0.093)

The mean and SD of the delayed kernel parameter estimates are:

	a		γ_1		τ	
	Mean	(SD)	Mean	(SD)	Mean	(SD)
Model A:	0.460	(0.037)	0.332	(0.072)	8.5×10^7	(5.4×10^7)
Model B:	0.461	(0.037)	0.332	(0.071)	0.3×10^7	(3.1×10^7)

The result for Model A excludes one large outlier where $a = 86.6$ and $\gamma_1 = 20.19$ (with corresponding delay parameter $\tau = 6 \times 10^7$).

Now we list the mean and SD of parameters estimated by the two joint models for scenario 2. Joint model 1 (JM1) has an instantaneous association and is incorrectly specified. Joint model 2 (JM2) has a cumulative association and is correctly specified. The true values of the parameters used to generate the data from joint model 1 are $\alpha = 0.25$ and $\gamma = 0.25$.

	$\alpha = 0.25$		$\gamma_1 = 0.25$	
	Mean	(SD)	Mean	(SD)
JM1 (incorrect):	0.686	(0.029)	0.208	(0.077)
JM2 (correct):	0.253	(0.010)	0.245	(0.080)

The estimated parameters of the delayed kernel models are:

	a		γ_1		τ	
	Mean	(SD)	Mean	(SD)	Mean	(SD)
Model A:	0.820	(0.047)	0.135	(0.067)	1.0×10^8	(0.6×10^8)
Model B:	0.820	(0.047)	0.128	(0.066)	2.7×10^6	(3.1×10^7)

The result for Model A excludes three outliers where $a = (186.0, 190.15, 193.45)$ and $\gamma = (177.2, 164.6, 171.8)$ (with corresponding delay parameters $\tau = (99.2, 177.0, 152.5)$). The result for Model B excludes one outlier where $a = 81.5$ and $\gamma = -1.68$ (with $\tau = 1.7 \times 10^8$).

S3.2.2 Prediction error. In Tables S1 and S2 we list the average prediction errors over the 50 iterations for the five models at the different base times for scenarios 1 and 2 respectively.

For both scenarios, the delayed kernel models perform similarly and have the highest prediction errors compared with the other models across all base times. However, they have more comparable performance to the other models at lower base times. Perhaps surprisingly, JM1 performs worse than JM2 and landmarking in both scenarios despite being correctly specified in scenario 1. JM2 performs best in scenario 1 while landmarking performs slightly better in scenario 2, despite JM2 being correctly specified in the latter.

Table S1. Average prediction error for simulated scenario 1 (instantaneous association) across the 50 iterations of the simulation.

	Average prediction error				
Base time	JM1 (correct)	JM2 (incorrect)	LM	Model A	Model B
1.5	0.177	0.169	0.174	0.185	0.186
3.5	0.185	0.162	0.167	0.221	0.223
5.5	0.172	0.154	0.157	0.212	0.214
7.5	0.167	0.152	0.160	0.203	0.205
9.5	0.162	0.149	0.155	0.192	0.193

Table S2. Average prediction error for simulated scenario 2 (cumulative association) across the 50 iterations of the simulation.

	Average prediction error				
Base time	JM1 (incorrect)	JM2 (correct)	LM	Model A	Model B
1.5	0.028	0.029	0.028	0.028	0.028
3.5	0.162	0.126	0.122	0.216	0.216
5.5	0.104	0.086	0.078	0.142	0.141
7.5	0.122	0.112	0.095	0.163	0.162
9.5	0.148	0.137	0.112	0.180	0.180

S4 R code for joint models

All joint models were fitted using the `R` package `JMbayes2`.[3] As default, `JMbayes2` uses 3 chains to check convergence during fitting. Errors are thrown if convergence is not reached. We used the default settings[3] for data sets with one longitudinal parameter and increased the number of iterations for multiple longitudinal variables (details are given below). All the data analysed in the main paper are available in the `JMbayes2` package:

1. PBC data
   (a) `pbc2` contains the PBC data set with time varying measurements of covariates
   (b) `pbc2.id` contains the PBC data set with only baseline covariate measurements per individual
2. AIDS data
   (a) `aids` contains the AIDS data set with time varying measurements of covariates
   (b) `aids.id` contains the AIDS data set with only baseline covariate measurements per individual
3. Liver data
   (a) `prothro` contains the Liver data set with time varying measurements of covariates
   (b) `prothros` contains the Liver data set with only baseline covariate measurements per individual.

The models fitted below are specified for the full data sets listed above. For the results presented in the main paper, the data sets were split into training and test data sets (using 10-fold cross validation) and the models were fitted to the training data at each iteration.

S4.1 PBC data

For the results in the main paper we treat the transplant event as a censoring event. To describe this we define a variable `status2` using

```
pbc2.id$status2 <- as.numeric(pbc2.id$status == "dead")
pbc2$status2 <- as.numeric(pbc2$status == "dead")
```

where `status2` $= 1$ if the individual's event is death and $= 0$ otherwise.

For the composite event (results shown in Section S5) we replace `status2` with `status3`,

```
pbc2.id$status3 <- as.numeric(pbc2.id$status != "alive")
pbc2$status3 <- as.numeric(pbc2$status != "alive")
```

defined as 1 if the individual experiences an event (death or a liver transplant) and 0 otherwise (still alive by end of study).

S4.1.1 Linear longitudinal model. Extract of `R` code used to fit the PBC data set using the simple linear model described in Section 4.2.1 in the main paper. Based on code in Rizopoulos (2012)[4] and Rizopoulos (2018):[5]

```
ctrl<-lmeControl(opt='optim', maxIter=500, msMaxIter = 500, msMaxEval = 500)
long.lin1<-lme(log(serBilir) ~ year, random = ~ year | id, data = pbc2,
                control = ctrl)
long.lin2<-lme(log(albumin) ~ year, random = ~ year | id, data = pbc2,
                control = ctrl)
long.lin3<-lme(log(prothrombin) ~ year, random = ~ year | id, data = pbc2,
                control = ctrl)
surv.pbc<-coxph(Surv(years, status2)~age, data=pbc2.id, model=TRUE)
JM.pbc.lin<-jm(surv.pbc, list(long.lin1, long.lin2, long.lin3),
            time_var = "year", n_iter = 12000L, n_burnin = 2000L, n_thin = 5L)
```

S4.1.2 Spline model. Extract of `R` code to fit the PBC data set using the natural cubic spline model described in Section 4.2.1 of the main paper. Based on code in Rizopoulos (2016)[6] and Rizopoulos (2018):[5]

```
long.sp1<-lme(log(serBilir) ~ ns(year,2,B=c(0,14.4)),
                random = ~ ns(year,2,B=c(0,14.4)) | id, data = pbc2,
                control = ctrl)
long.sp2<-lme(log(albumin) ~ ns(year,2,B=c(0,14.4)),
                random = ~ ns(year,2,B=c(0,14.4)) | id, data = pbc2,
                control = ctrl)
long.sp3<-lme(log(prothrombin) ~ ns(year,2,B=c(0,14.4)),
```

```
                random = ~ ns(year,2,B=c(0,14.4)) | id, data = pbc2,
                control = ctrl)
surv.pbc<-coxph(Surv(years, status2)~age, data=pbc2.id, model=TRUE)
JM.pbc.sp<-jm(surv.pbc, list(long.sp1, long.sp2, long.sp3),
           time_var = "year", n_iter = 12000L, n_burnin = 2000L, n_thin = 5L)
```

Based on code from Rizopolous (2018)[5], for the PBC data we used 3 chains, 12000 iterations, a burn-in of 2000, and a thinning factor of 5. No convergence errors were thrown when implementing the analysis.

S4.2 AIDS data

Extract of `R` code to fit the AIDS data set using the model described in Section 4.3.1 of the main paper. Based on code in Section 4.2 of Rizopoulos (2012):[4]

```
long.aids<-lme(CD4~obstime+obstime:drug, random=~obstime|patient, data=aids)
surv.aids<-coxph(Surv(Time, death)~drug+prevOI+AZT+gender, data=aids.id,
                 x=TRUE)
JM.aids<-jm(surv.aids, long.aids, time_var="obstime")
```

For the AIDS data we used the default settings in `JMbayes2`: 3 chains, 3000 iterations, and a burn-in of 500. No convergence errors were thrown during the analysis.

S4.3 Liver data

Extract of `R` code to fit the Liver data set using the model described in Section 4.4.1 of the main paper. Based on code in Section 5.1.2 of Rizopoulos (2012):[4]

```
prothro$t0<-as.numeric(prothro$time==0)
long.proth<-lme(pro~treat*(ns(time, 3) + t0),
                random=list(id=pdDiag(form=~ns(time,3))), data = prothro)
surv.proth<-coxph(Surv(Time, death)~treat, data=prothros, x=TRUE)
JM.proth<-jm(surv.proth, long.proth, time_var="time")
```

For the Liver data we used the default settings in `JMbayes2`: 3 chains, 3000 iterations, and a burn-in of 500. No convergence errors were thrown during the analysis.

S5 PBC data with composite event

In the main paper we present the results for the PBC data set for models that treat death as the event of interest and transplant events as censoring events. Here we show the results for models that treat the two events (death or transplant) as a single composite event. Figure S7 shows the result for a fixed base time $t = 3$ years and varying prediction time u. Figure S8 shows the results for three fixed prediction windows and varying base time t. With comparison to Figures 5 and 6 in the main paper, we see that the delayed kernel models perform more similarly to the joint models for the composite event analysis (prediction error is slightly higher overall).

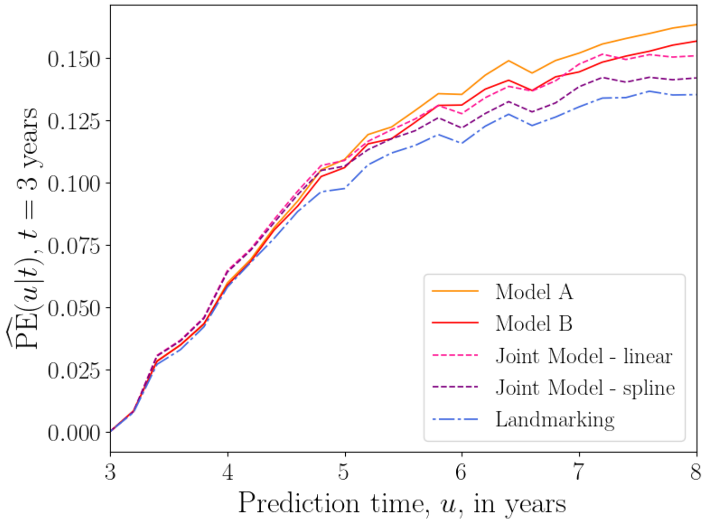

Figure S7. Fixed base time results for the PBC data with models fitted treating the two events (death and transplant) as a single composite event. The plot shows overall prediction error $\widehat{\mathrm{PE}}(u|t)$ as a function of prediction time u (in years) with fixed base time $t = 3$ years. Prediction error is calculated for u values from 3 to 8 years, with 0.2 year increments. A squared loss function was used in Equation (26) in the main paper. The prediction error plotted at each time u is from a 10-fold cross validation analysis. The results from models A and B of the delayed kernel approach are plotted alongside the landmarking model and two joint models (one that uses a linear longitudinal model for the time-dependent covariates, and another that uses cubic splines). Other than the definition of the composite event, the models fitted are the same as those described in the main paper.

S6 Evaluating prediction error for JM and LM models

The definition of prediction error $\widehat{\mathrm{PE}}(u|t)$ is given in Equation (26) of the main paper. This is identical to the equation for prediction error quoted on pg. 34 in Rizopoulos (2016).[6] For delayed kernel models A and B prediction error is calculated using a `Python` code that exactly follows this equation.

The `JMbayes2` package provides the function `tvBrier` to calculate prediction error for joint models (equivalent to `prederrJM` in `JMbayes` described in Rizopoulos (2016)[6]). In `JMbayes`, the function `prederrJM` can be used for standard Cox models and, therefore, landmarking models. However, the source code for `tvBrier` and `prederrJM` varies very slightly from Equation (26). Specifically,

1. they use $\sum_{i;T_i>t}$ instead of the $\sum_{i;T_i\geq t}$ in Equation (26),
2. for the first term (individuals who are still alive), they specify the condition $I(T_i > u)$ instead of $I(T_i \geq u)$,
3. and for the second term (individuals who have experienced the event), `prederrJM` specifies $\delta_i I(T_i \leq u)$ instead of $\delta_i I(T_i < u)$. (NB: `tvBrier` uses the latter).

These inconsistencies only have an effect when u or t are exactly equal to one (or more) of the event times T_i in the test data. In the PBC and Liver data sets, event times T_i are quoted to a large number of decimal places meaning we never encounter $u = T_i$ or $t = T_i$ (since we vary t and u in steps of 0.2). However, for the AIDS data set, event times are stored to a lower number of decimal places and we do encounter $u = T_i$ or $t = T_i$ for some values of t and u. For the joint model and landmarking results presented in the main paper we use edited versions of `tvBrier` and `prederrJM` where inequalities exactly match Equation (26) (and hence the equation for prediction error in Rizopoulos (2016)[6]). This code can be found at the GitHub repository https://github.com/AnnieDavies/Supplement_Davies_Coolen_Galla_2023. For the PBC and Liver data sets the results in the main paper are the same as those using the `tvBrier` and `prederrJM` code without these changed inequalities. Figures S9 and S10 show the results for the AIDS data without these changes. Comparing these to Figures 7 and 8 in the main paper, it is clear the effect of these changes is very minor.

The handling of exceptions in `tvBrier` and `prederrJM` is such that the function generates an output `NA` if no-one experiences a (non-censoring) event in the window $[t, u]$. Because we are splitting the data sets randomly into training and test sets at different iterations, we occasionally encounter this scenario for certain windows. If there are no non-censoring events in a given window $[t, u]$, the second term in Equation (26) is equal to zero. Therefore, we edited the `tvBrier` and `prederrJM` source code to handle this scenario (see the Github repository https://github.com/AnnieDavies/Supplement_Davies_Coolen_

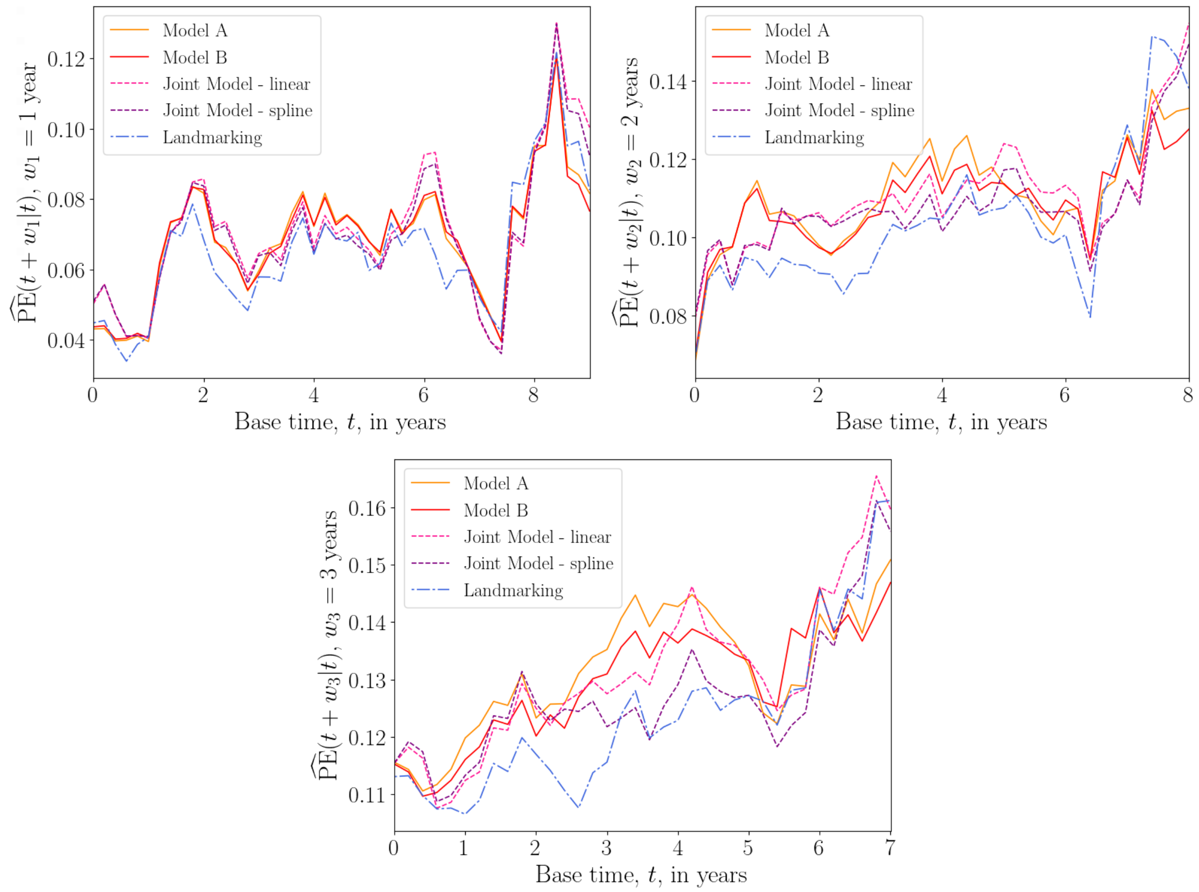

Figure S8. Fixed prediction window results for the PBC data with models fitted treating the two events (death and transplant) as a single composite event. Plots show overall prediction error $\widehat{\text{PE}}(u|t)$ versus base time t (in years), with prediction windows $w_1 = 1$ year, $w_2 = 2$ years and $w_3 = 3$ years. The prediction times are $u = t + w$. The prediction error is calculated for t ranging from 0 to 9,8 or 7 years for w_1, w_2 and w_3 respectively, with 0.2 year increments. A squared loss function was used in Equation (26) in the main paper. The prediction error plotted at each time t is from a 10-fold cross validation analysis. Results from models A and B of the delayed kernel approach are plotted alongside the landmarking model and two joint models; one that uses a linear longitudinal model for the time-dependent covariates, and another that uses cubic splines. Other than the definition of the composite event, the models fitted are the same as those described in the main paper.

Galla_2023). The results in the main paper are for this edited code. Compared to the original codes, these edits have a negligible effect on results.

For the version of `prederrJM` for Cox models, we also obtain an output of `NA` if there is no-one censored in the interval $[t, u]$. In `tvBrier` (for joint models) this is handled by including the argument `na.rm=TRUE` when we perform the sum $\sum_{i;T_i \geq t}$. We therefore added this argument to the function for Cox models.

All changes made to source code were very minor and had an almost negligible effect on all results. Changes were made to the code only to ensure that all models were evaluated with exactly the same prediction error equation consistent with the equation quoted in literature.[2,6,7]

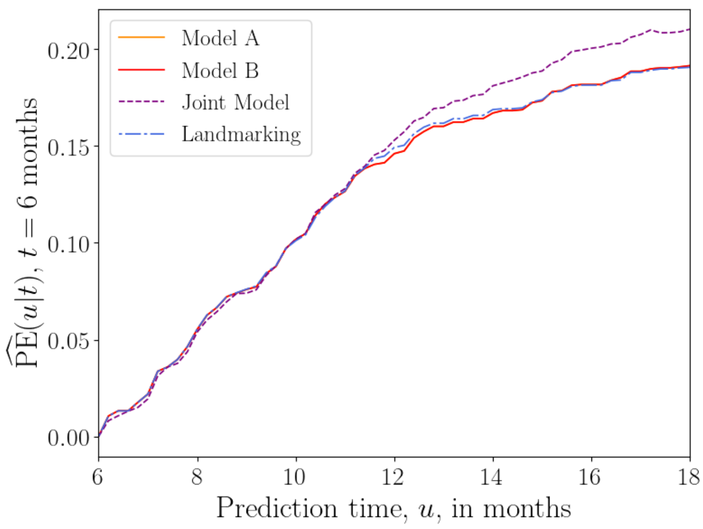

Figure S9. Fixed base time results for the AIDS data set using the `prederrJM` code (for the joint model and landmarking model) without changes made to the inequalities. Overall prediction error $\widehat{\mathrm{PE}}(u|t)$ plotted versus prediction time u (in months) for the AIDS data with fixed base time $t = 6$ months. This error is calculated for u ranging from 6 to 18 months, at 0.2 month intervals. In Equation (26) in the main paper a squared loss function was used. The prediction error plotted at each time u is from a 10-fold cross validation analysis. The results from delayed kernel models A and B are plotted alongside the results from the landmarking model and a joint model. The results from model A (orange line) cannot be seen because they overlap with the results from model B (red line).

S7 Results with decaying association parameter at s=0

The association kernels $\beta_\mu(t, t', s)$ for models A and B as specified in Equations (16) and (17) of the main paper do not hold for $s = 0$. In the data sets we analyse, some individuals are observed only once meaning their final observation time is $s = 0$. For the results presented in the main paper we treat the association parameter of these individuals as fixed, $\beta_\mu(t) = a_\mu$. Another option is to define a decaying parameter, $\beta_\mu(t) = a_\mu \mathrm{e}^{-t/\tau_\mu}$. The results for this latter choice are shown in Figures S11 and S12 for the PBC data (treating transplants as a censoring event), in Figures S13 and S14 for the AIDS data, and in Figures S15 and S16 for the Liver data. For the PBC and Liver data, the results with $\beta_\mu(t) = a_\mu \mathrm{e}^{-t/\tau_\mu}$ and $\beta_\mu(t) = a_\mu$ are similar when the base time $t \gtrsim 1$ year. When we restrict the individuals in the test data to having observations over a smaller time frame, the prediction error for these models is much larger. This effect is increased for the larger prediction windows. This can be understood because for smaller t many individuals in the test data will have been observed only once and the parameter $\beta_\mu(t) = a_\mu \mathrm{e}^{-t/\tau_\mu}$ means the effect of this observation is decayed at later times. Similarly in the AIDS data set, the results are similar to the results in the main paper except when the individuals are restricted to only one observation (at $t = 0$). Perhaps another reasonable choice of association parameter for $s = 0$ is a hybrid of the fixed and decaying association, e.g. $\beta_\mu(t) = a_\mu(1 + \mathrm{e}^{-t/\tau_\mu})$.

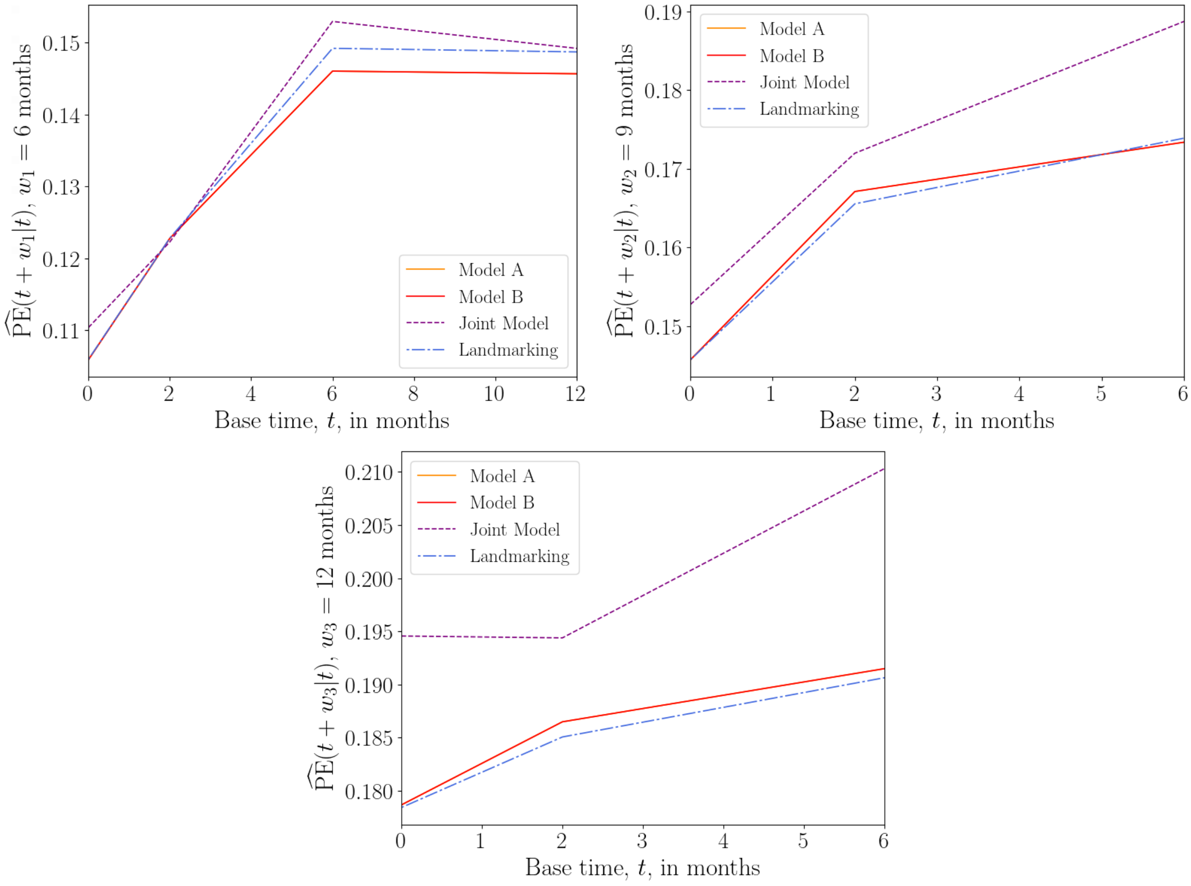

Figure S10. Fixed prediction window results for the AIDS data set using the `prederrJM` code (for the joint model and landmarking model) without changes made to the inequalities. Overall prediction error $\widehat{\mathrm{PE}}(u|t)$ versus base time t (in months) for the AIDS data with three fixed prediction windows: $w_1 = 6$ months, $w_2 = 9$ months and $w_3 = 12$ months. The prediction times are $u = t + w$. Observations are made at times $0, 2, 6, 12, 18$ months for all individuals in this data set. Prediction errors are hence only updated at these time points. For prediction window w_1, prediction error is measured for $t = 0, 2, 6$ and 12 months. For windows w_2 and w_3, the error is measured at $t = 0, 2$ and 6 months only. In Equation (26) in the main paper we used a squared loss function. The prediction error plotted at each time t is from a 10-fold cross validation analysis. The results from delayed kernel models A and B are plotted alongside the landmarking model and a joint model. The results from model A (orange line) cannot be seen clearly because they overlap with the results from model B (red line).

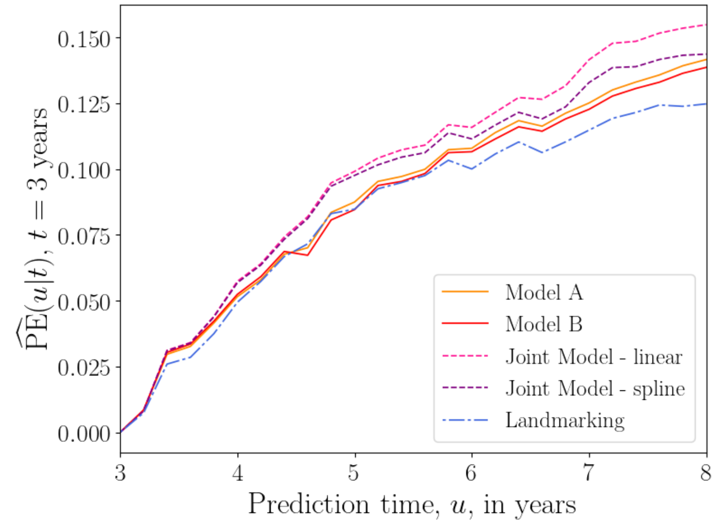

Figure S11. Fixed base time results for the PBC data with a decaying association in models A and B, $\beta_\mu(t) = a_\mu \mathrm{e}^{-t/\tau_\mu}$, for individuals who have their final observation time $s = 0$. The plot shows overall prediction error $\widehat{\mathrm{PE}}(u|t)$ as a function of prediction time u (in years) with fixed base time $t = 3$ years. Prediction error is calculated for u values from 3 to 8 years, with 0.2 year increments. A squared loss function was used in Equation (26) in the main paper. The prediction error plotted at each time u is from a 10-fold cross validation analysis. The results from models A and B of the delayed kernel approach are plotted alongside the landmarking model and two joint models (one that uses a linear longitudinal model for the time-dependent covariates, and another that uses cubic splines). Other than the definition of the association for $s = 0$ in models A and B, the models fitted are the same as those described in the main paper (i.e. we treat transplant events as a censoring event).

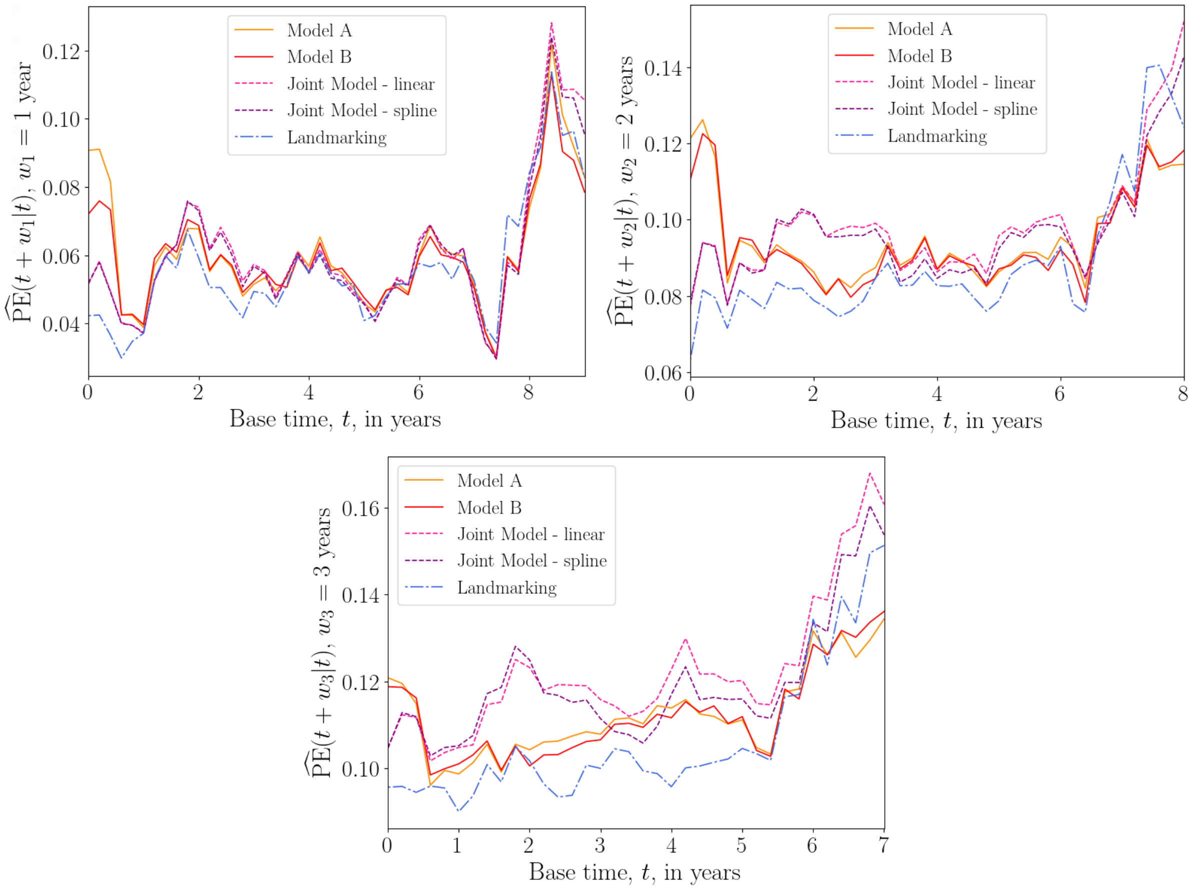

Figure S12. Fixed prediction window results for the PBC data with a decaying association in models A and B, $\beta_\mu(t) = a_\mu e^{-t/\tau_\mu}$, for individuals who have their final observation time $s = 0$. Plots show overall prediction error $\widehat{\mathrm{PE}}(u|t)$ versus base time t (in years), with prediction windows $w_1 = 1$ year, $w_2 = 2$ years and $w_3 = 3$ years. The prediction times are $u = t + w$. The prediction error is calculated for t ranging from 0 to 9,8 or 7 years for w_1, w_2 and w_3 respectively, with 0.2 year increments. A squared loss function was used in Equation (26) in the main paper. The prediction error plotted at each time t is from a 10-fold cross validation analysis. Results from models A and B of the delayed kernel approach are plotted alongside the landmarking model and two joint models; one that uses a linear longitudinal model for the time-dependent covariates, and another that uses cubic splines. Other than the definition of the association for $s = 0$ in models A and B, the models fitted are the same as those described in the main paper (i.e. we treat transplant events as a censoring event).

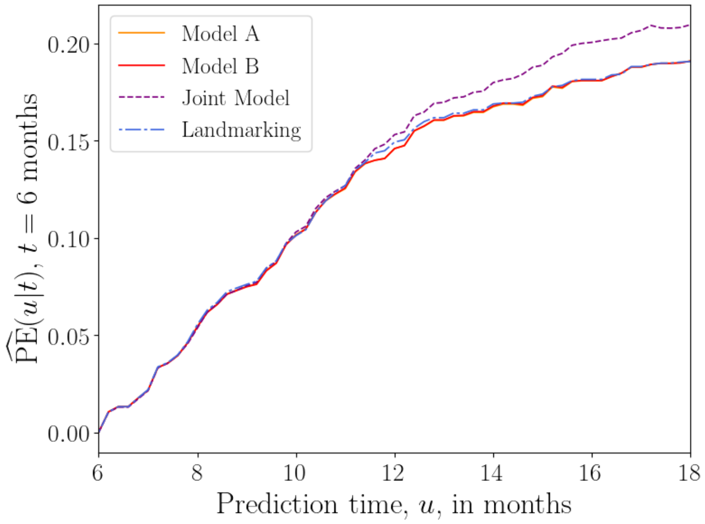

Figure S13. Fixed base time results for the AIDS data with a decaying association in models A and B, $\beta_\mu(t) = a_\mu e^{-t/\tau_\mu}$, for individuals who have their final observation time $s = 0$. Overall prediction error $\widehat{\mathrm{PE}}(u|t)$ plotted versus prediction time u (in months) with fixed base time $t = 6$ months. This error is calculated for u ranging from 6 to 18 months, at 0.2 month intervals. In Equation (26) in the main paper a squared loss function was used. The prediction error plotted at each time u is from a 10-fold cross validation analysis. The results from delayed kernel models A and B are plotted alongside the results from the landmarking model and a joint model. Other than the definition of the association for $s = 0$ in models A and B, the models fitted are the same as those described in the main paper. The results from model A (orange line) cannot be seen because they overlap with the results from model B (red line).

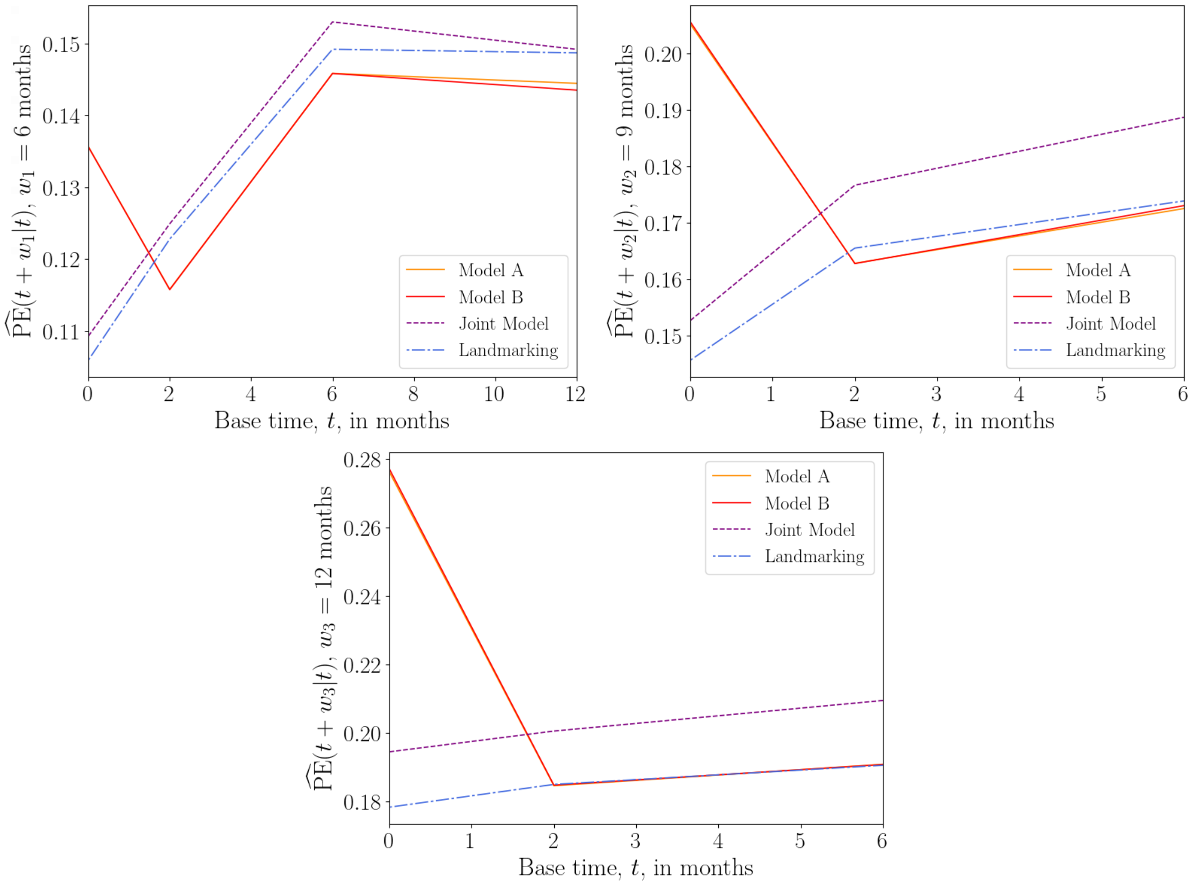

Figure S14. Fixed prediction window results for the AIDS data with a decaying association in models A and B, $\beta_\mu(t) = a_\mu e^{-t/\tau_\mu}$, for individuals who have their final observation time $s = 0$. Overall prediction error $\widehat{\mathrm{PE}}(u|t)$ versus base time t (in months) with three fixed prediction windows: $w_1 = 6$ months, $w_2 = 9$ months and $w_3 = 12$ months. The prediction times are $u = t + w$. Observations are made at times $0, 2, 6, 12, 18$ months for all individuals in this data set. Prediction errors are hence only updated at these time points. For prediction window w_1, prediction error is measured for $t = 0, 2, 6$ and 12 months. For windows w_2 and w_3, the error is measured at $t = 0, 2$ and 6 months only. In Equation (26) in the main paper we used a squared loss function. The prediction error plotted at each time t is from a 10-fold cross validation analysis. The results from delayed kernel models A and B are plotted alongside the landmarking model and a joint model. Other than the definition of the association for $s = 0$ in models A and B, the models fitted are the same as those described in the main paper. The results from model A (orange line) cannot be seen clearly because they overlap with the results from model B (red line).

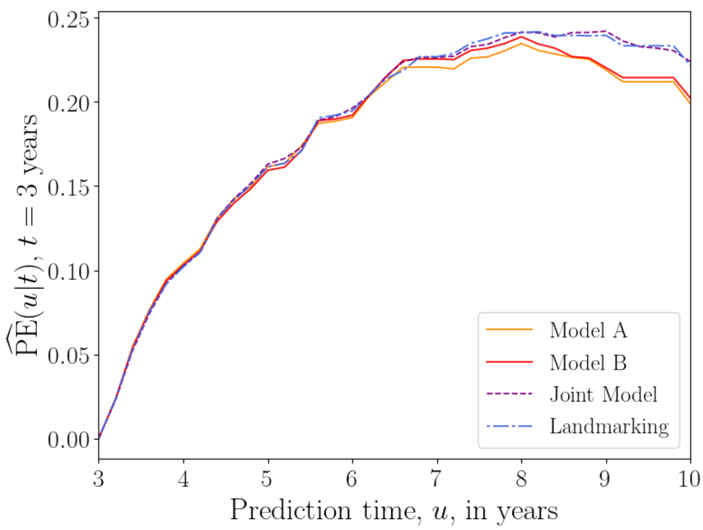

Figure S15. Fixed base time results for the Liver data with a decaying association in models A and B, $\beta_\mu(t) = a_\mu \mathrm{e}^{-t/\tau_\mu}$, for individuals who have their final observation time $s = 0$. Overall prediction error $\widehat{\mathrm{PE}}(u|t)$ plotted versus prediction time u (in years) with fixed base time $t = 3$ years. This error is calculated for u ranging from 3 to 10 years, with 0.2 year increments. In Equation (26) in the main paper we used a squared loss function. The prediction error plotted at each time u is from a 10-fold cross validation analysis. The results from delayed kernel models A and B are plotted alongside the results from the landmarking model and a joint model. Other than the definition of the association for $s = 0$ in models A and B, the models fitted are the same as those described in the main paper.

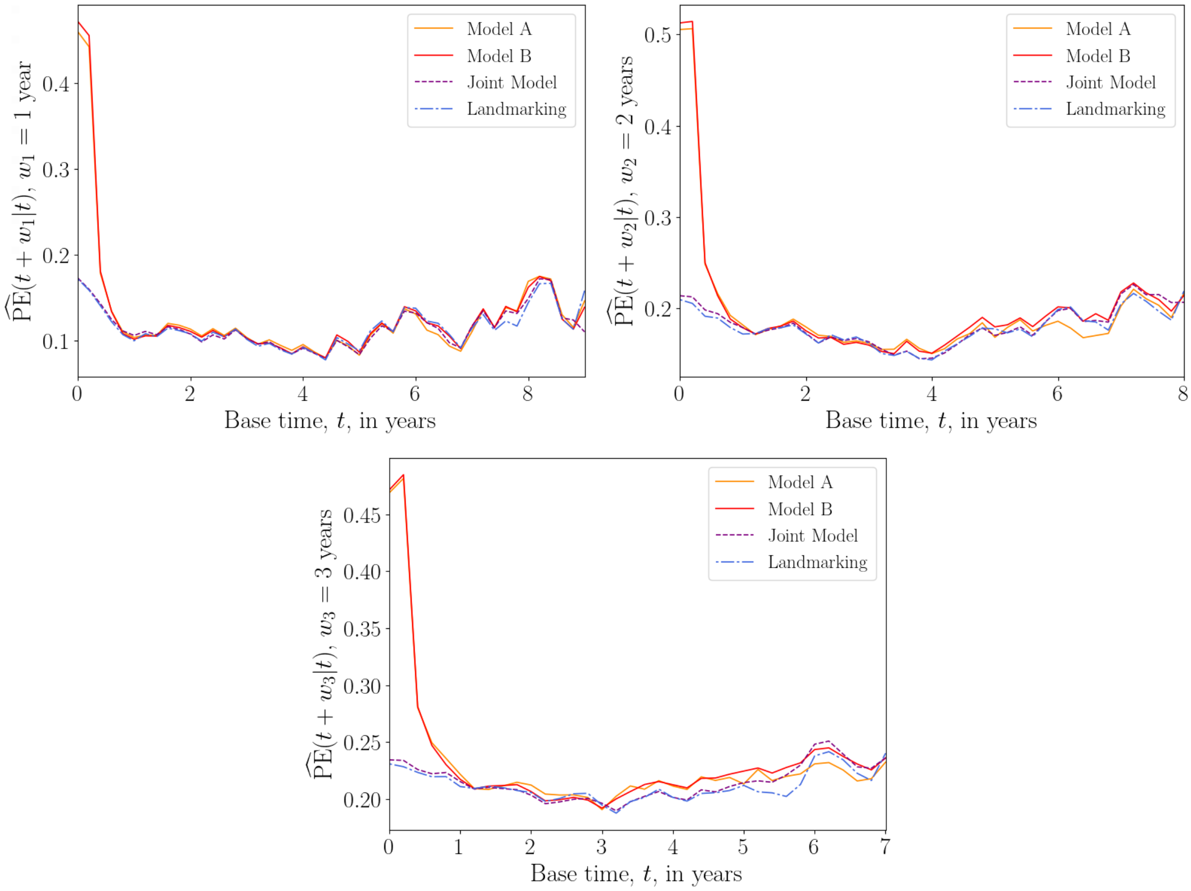

Figure S16. Fixed prediction window results for the Liver data with a decaying association in models A and B, $\beta_\mu(t) = a_\mu \mathrm{e}^{-t/\tau_\mu}$, for individuals who have their final observation time $s = 0$. Overall prediction error $\widehat{\mathrm{PE}}(u|t)$ plotted against base time t (in years) for the Liver data with three fixed prediction windows, $w_1 = 1$ year, $w_2 = 2$ years and $w_3 = 3$ years. The prediction times are $u = t + w$. The error is calculated for t ranging from 0 to 9,8 or 7 years, for w_1, w_2 and w_3 respectively, with 0.2 year intervals. In Equation (26) in the main paper a squared loss function was used. The prediction error plotted at each time t is from a 10-fold cross validation analysis. The results from delayed kernel models A and B are plotted alongside the landmarking model and a joint model. Other than the definition of the association for $s = 0$ in models A and B, the models fitted are the same as those described in the main paper.

References

1. Breslow NE. Discussion on Professor Cox's paper. *J Roy Stat Soc B Met* 1972; 34: 216–217.
2. Rizopoulos D, Molenberghs G and Lesaffre EMEH. Dynamic predictions with time-dependent covariates in survival analysis using joint modeling and landmarking. *Biometrical J* 2017; 59(6): 1261–1276.
3. Rizopoulos D, Papageorgiou G and Afonso PM. Jmbayes2: Extended joint models for longitudinal and time-to-event data, version 0.4-0. CRAN, 2023. Available at: https://CRAN.R-project.org/package=JMbayes2.
4. Rizopoulos D. *Joint models for longitudinal and time-to-event data with applications in R*. CRC Biostatistics Series, New York: Chapman and Hall, 2012.
5. Rizopoulos D. Multivariate joint models vignette. Online, 2018. Accessed 07/07/21 from http://www.drizopoulos.com/vignettes/multivariate%20joint%20models.
6. Rizopoulos D. The R package JMbayes for fitting joint models for longitudinal and time-to-event data using MCMC. *J Stat Softw* 2016; 72(7): 1–46.
7. Henderson R, Diggle P and Dobson A. Identification and efficacy of longitudinal markers for survival. *Biostatistics* 2002; 3(1): 33–50.
